# Alterations in the molecular control of mitochondrial turnover in COPD lung and airway epithelial cells

**DOI:** 10.1038/s41598-024-55335-8

**Published:** 2024-02-27

**Authors:** Christy B. M. Tulen, Cheryl van de Wetering, Caspar H. J. Schiffers, Ellen Weltjens, Birke J. Benedikter, Pieter A. Leermakers, Juliana H. Boukhaled, Marie-José Drittij, Bernd T. Schmeck, Niki L. Reynaert, Antoon Opperhuizen, Frederik-Jan van Schooten, Alexander H. V. Remels

**Affiliations:** 1 School of Nutrition and Translational Research in Metabolism (NUTRIM), Department of Pharmacology and Toxicology, Maastricht University Medical Center+, Universiteitssingel 50, 6629 ER Maastricht, The Netherlands; 2 School of Nutrition and Translational Research in Metabolism (NUTRIM), Department of Respiratory Medicine, Maastricht University Medical Center, Maastricht, The Netherlands; 3 School of Nutrition and Translational Research in Metabolism (NUTRIM), Department of Microbiology, Maastricht University Medical Center, Maastricht, The Netherlands; 4 Institute for Lung Research, Philipps-University Marburg, Marburg, Germany; 5 Department for Respiratory and Critical Care Medicine, Clinic for Respiratory Infections, University Medical Center Marburg, Marburg, Germany; 6 German Centers for Lung Research (DZL) and for Infectious Disease Research (DZIF), SYNMIKRO Center for Synthetic Microbiology, Philipps-University Marburg, 35037 Marburg, Germany; 7 Primary Lung Culture (PLUC) Facility, Maastricht University Medical Center, Maastricht, The Netherlands; 8 Office of Risk Assessment and Research, Netherlands Food and Consumer Product Safety Authority (NVWA), Utrecht, The Netherlands; 9 Member of the German Center for Lung Research (DZL), Universities of Giessen and Marburg Lung Center, Giessen, Germany

**Keywords:** Chronic obstructive pulmonary disease, Peripheral lung tissue, Primary bronchial epithelial cells, Mitochondrial biogenesis, Mitophagy, Chronic obstructive pulmonary disease, Mechanisms of disease, Cell biology, Molecular biology

## Abstract

Abnormal mitochondria have been observed in bronchial- and alveolar epithelial cells of patients with chronic obstructive pulmonary disease (COPD). However, it is unknown if alterations in the molecular pathways regulating mitochondrial turnover (mitochondrial biogenesis *vs* mitophagy) are involved. Therefore, in this study, the abundance of key molecules controlling mitochondrial turnover were assessed in peripheral lung tissue from non-COPD patients (n = 6) and COPD patients (n = 11; GOLDII n = 4/11; GOLDIV n = 7/11) and in both undifferentiated and differentiated human primary bronchial epithelial cells (PBEC) from non-COPD patients and COPD patients (n = 4–7 patients/group). We observed significantly decreased transcript levels of key molecules controlling mitochondrial biogenesis (*PPARGC1B*, *PPRC1*, *PPARD*) in peripheral lung tissue from severe COPD patients. Interestingly, mRNA levels of the transcription factor *TFAM* (mitochondrial biogenesis) and *BNIP3L* (mitophagy) were increased in these patients. In general, these alterations were not recapitulated in undifferentiated and differentiated PBECs with the exception of decreased *PPARGC1B* expression in both PBEC models. Although these findings provide valuable insight in these pathways in bronchial epithelial cells and peripheral lung tissue of COPD patients, whether or not these alterations contribute to COPD pathogenesis, underlie changes in mitochondrial function or may represent compensatory mechanisms remains to be established.

## Introduction

Chronic obstructive pulmonary disease (COPD) is one of the leading causes of morbidity and mortality globally^1,2^. Classification of COPD disease severity is based on the degree of airway obstruction that results from pathological features in the bronchial compartment (chronic bronchitis) and the parenchyma (emphysema)^3,4^.

Inhalation of cigarette smoke (CS) and other noxious particles is the main risk factor for developing COPD^5^. After inhalation, toxicants and particles first reach the epithelial layer of the bronchial compartment where they can mediate damage and stress responses^6^. Changes in the composition and functionality of the bronchial epithelium in COPD patients (smokers) include basal- and goblet cell hyperplasia and aberrant number/length of ciliated cells^7–14^. During the development of COPD, however, not only the bronchial compartment but also the smaller airways and alveoli, are affected^15,16^.

In addition to structural- and functional changes described above, COPD is also characterized by aberrant cellular processes in epithelial cells of the airways and lungs including (chronic) inflammation cell death and oxidative stress^16^.

It has been shown that mitochondria, besides their role in energy metabolism, are critical regulators of these cellular processes^17,18^. Interestingly, abnormalities at the level of the mitochondrion have been described in COPD. Indeed, previous studies showed elongated and swollen mitochondria as well as fragmentation, branching and cristae depletion in primary bronchial epithelial cells (PBEC) from ex-smoking COPD patients with very severe disease compared to never-smoker controls^19^, and in PBECs from COPD patients relative to smokers without COPD^20–22^. Data on mitochondria in peripheral lung tissue of COPD vs non-COPD patients, likely due to the difficulty associated with obtaining parenchymal lung tissue, is almost non-existent. These changes are likely associated with exposure to CS as it has been convincingly shown that CS exposure of both bronchial-, small airway- and alveolar epithelial cells results in similar abnormalities in mitochondrial structure/phenotype^19,20,23,24^.

Mitochondria are dynamic organelles that are capable to adapt their morphology and energy production to meet demand. Mitochondrial homeostasis is preserved by the interplay between the synthesis of new organelles (mitochondrial biogenesis), and breakdown of damaged ones (mitophagy). Mitochondrial dynamics, controlled by fusion and fission events), is a key driver for these processes^18,25^.

Mitochondrial biogenesis is tightly regulated by transcriptional coactivators of the Peroxisome Proliferator-Activated Receptor (PPAR) Gamma (PPARG), Coactivator 1 (PPARGC1) family, respectively PPARGC1 Alpha (PPARGC1A), PPARGC1 Beta (PPARGC1B) and PPARG Coactivator-Related 1 (PPRC1), and their downstream transcription factors such as Nuclear Respiratory Factor 1 (NRF1), Nuclear Respiratory Factor 2 (NRF2) and Transcription Factor A, Mitochondrial (TFAM)^26–28^.

On the other side of the balance, mitochondrial quality control is mediated by the selective degradation of damaged or defective mitochondria by ubiquitin- or receptor-mediated mitophagy. Ubiquitin-mediated mitophagy is triggered by a loss of the mitochondrial membrane potential leading to stabilization and accumulation of PTEN-Induced Kinase 1 (PINK1) on the outer mitochondrial membrane. Subsequently, recruitment of Parkin RBR E3 Ubiquitin Protein Ligase (PRKN) to the mitochondria results in ubiquitination of mitochondrial proteins triggering the formation of an autophagosome and degradation of the organelle.

Receptor-mediated mitophagy is mediated by receptor proteins anchored on cytosolic side of the outer mitochondrial membrane, such as BCL2 Interacting Protein 3 (BNIP3), BNIP3-Like (BNIP3L) and FUN14 Domain Containing 1 (FUNDC1). These membrane receptors interact directly with Microtubule-Associated Protein 1 Light Chain 3 (MAP1LC3) Beta on the autophagosomal membrane, making ubiquitination redundant. In both mitophagy pathways, ultimately, specific general autophagy proteins are required to facilitate encapsulation of the damaged or defective mitochondria in an autophagosome^29–31^.

Cellular stress, as induced by for example inhaled CS, can result in alterations in mitochondrial bioenergetics and can induce mitochondrial dysfunction^18^^,^^32–34^.

Although previous studies have convincingly shown abnormal mitochondrial morphology/content in COPD, studies comprehensively assessing the molecular pathways controlling this are lacking. Moreover, although some studies have tried to address this in PBECs of COPD vs non-COPD patients, data on these pathways in peripheral lung tissue is almost non-existent.

Therefore, in the current study, our primary aim was to assess the abundance of essential regulators involved in mitochondrial turnover in lung tissue and cells of the airways from non-COPD patients and COPD patients at different stages of the disease. Biological material assessed in our analyses included peripheral lung tissue as well as isolated PBEC that were either cultured submerged (undifferentiated; basal cells) or at air–liquid interface (ALI) (differentiated; ciliated cells, secretory club cells, mucus-producing goblet cells). We hypothesized alterations in the abundance of key constituents associated with mitochondrial biogenesis, mitophagy, dynamics and metabolism in peripheral lung tissue as well as in bronchial epithelial cells from COPD patients compared to non-COPD patients.

## Materials and methods

All methods were performed in accordance with the relevant guidelines and regulations.

### Human peripheral lung tissue collection

Lung tissue was obtained from 7 patients with very severe emphysema (GOLDIV) who underwent lung volume reduction surgery, and had quit smoking for at least 6 months before the surgery. These patients were prescribed combination therapy of inhaled corticosteroids and long-acting β2-agonists, tiotropium/ipratropiumbromide and salbutamol, on demand. In addition, lung tissue was obtained from 6 control subjects with normal lung function (without cough and/or sputum production), and 4 moderate (GOLDII) COPD patients undergoing resection of a solitary peripheral tumor. For all subjects, a history of respiratory diseases other than COPD as well as increased respiratory complaints or respiratory tract infection during the 4 weeks preceding the study were considered as criteria for exclusion. The study was approved by the medical ethical committee of the Maastricht University Medical Center+ (MUMC+) (MEC 02-043.3). All subjects gave their informed consent in writing. Patient characteristics and clinical information are shown in Table 1.

**Table 1:** Subject characteristics: peripheral lung tissue from non-COPD patients and COPD patients.

Characteristics	Non-COPD patients	COPD patients		
		COPD patients	GOLDII	GOLDIV
Number of subjects	6	11	4/11	7/11
Male/female	4/2	5/6	4/-	1/6
Age (years)	65.2 ± 1.9^§^	59.3 ± 3.4	69.8 ± 4.2	53.3 ± 3.0*
Body mass index	24.2 ± 2.0	24.7 ± 1.3	24.8 ± 2.6	24.7 ± 1.5
Smoking behaviour (Ex-/current smoker)	5/1	7/4	-/4	7/-
Pack-years (years)	31.6 ± 6.9^§^	34.5 ± 4.7^§^	29.5 ± 7.8	37.8 ± 6.0^$^
FEV_1_ (absolute) (L)	2.6 ± 0.3	1.1 ± 0.2**	2.1 ± 0.2	0.6 ± 0.05**
FVC (absolute) (L)	3.2 ± 0.4	2.7 ± 0.2	3.6 ± 0.2	2.2 ± 0.2*
FEV_1_/FVC (%)	83.1 ± 2.8	39.0 ± 5.1**	59.2 ± 3.6**	27.5 ± 2.2**
Diffusing capacity of carbon monoxide (DLCO) (%)	90.8 ± 9.4^§^	52.9 ± 7.6^‡^*	70.6 ± 9.1	38.8 ± 6.8^‡^**

Characteristics of non-COPD patients and COPD patients in different disease stages from who peripheral lung tissue was investigated. Data are presented as mean ± SEM.

*FEV*_*1*_ forced expiratory volume in first second, *FVC* forced vital capacity.

Statistical significance is indicated as *p < 0.05 and **p < 0.01 compared to non-COPD patients.

^a^1 missing value, ^b^2 missing values. Statistical differences between COPD patients *versus* non-COPD patients (two groups) were tested using a two-tailed unpaired parametric t-test (normal distribution), or an unpaired nonparametric Mann–Whitney test (non-normal distribution). In addition, in case of testing the difference between COPD GOLDII or GOLDIV patients *versus* non-COPD patients (three groups), we used a one-way ANOVA and a Dunnett’s post-hoc test for multiple comparisons (normal distribution).

### Undifferentiated PBEC: collection, isolation and proliferation

Lung tissue used for the isolation of PBEC was obtained from the Maastricht Pathology Tissue Collection (MPTC). Collection, storage and use of tissue and patient data were performed in agreement with the “Code for Proper Secondary Use of Human Tissue in the Netherlands” (http://www.fmwv.nl). The scientific board of the MPTC approved the use of materials for this study under MPTC2010-019. In addition, the local medical ethical committee approved the use of human tissue (METC 2017-0087).

Isolation, culture and characterization of cells was performed by the Primary Lung Culture (PLUC) facility at the MUMC+ as previously described^35,36^. The PLUC facility provided PBEC isolated from resected bronchus rings remote from the diseased areas of patients undergoing lung surgery due to lung cancer at MUMC+ . PBEC from 7 donors without known history of COPD and 4 donors with known history of COPD (GOLDII) were provided for this study. Characteristics of the subject are summarized in Table S1.

PBEC of 11 donors (5 × 10^5^ cells, passage 1) were thawed, seeded and expanded in T75-flasks (passage 2; Corning Costar) on pre-coated growth areas (coating: 10 μg/mL human fibronectin, 30 μg/ml PureCol Type I Collagen Solution (Bovine) and 10 μg/mL bovine serum albumin (BSA) diluted in Hank’s Balanced Salt Solution (HBSS; no calcium, no magnesium, no phenol red) (Gibco)) in Lonza Bronchial Epithelial Basal Medium supplemented with Bronchial Epithelial Cell Growth Medium (BEGM) singlequots (except Gentamycin) (Lonza), BSA (1.5 µg/mL) and 1% penicillin/streptomycin (Gibco) (i.e., BEGM complete). After medium refreshment every other day, confluence was reached upon 5–6 days of proliferation. Thereafter, PBEC were trypsinized, counted and seeded in quintuplicate in pre-coated 6 wells, 12 wells or 60 mm dishes in BEGM complete medium. Medium was refreshed every other day. Upon 85% confluence, PBEC were starved overnight using Lonza Bronchial Epithelial Basal Medium supplemented with Bronchial Epithelial Cell Growth Medium singlequots (except gentamycin, bovine pituitary extract and epidermal growth factor) (Lonza), BSA (1.5 µg/mL) and 1% penicillin/streptomycin (Gibco) (i.e., BEGM starvation). The next day, after 24 h of starvation, undifferentiated PBEC were harvested in appropriate buffers for follow-up analysis.

### Differentiated PBEC: collection, isolation, proliferation and differentiation

PBEC from COPD patients (n = 6) and control subjects (n = 6) were collected at the University Medical Center Marburg in agreement with local ethics regulations (Marburg 223/12) (code 223/12). Briefly, bronchoscopies were conducted according to American Thoracic Society consensus procedure. During bronchoscopy, bronchial brushings were collected using protected specimen brushes. Cells were scraped from the brush and incubated in 8 mL accutase with gentle agitation for 2 min before 8 min centrifugation at 300 rcf. Cells were resuspended in Airway Epithelial Cell Growth Medium (AEGM, Promocell) and seeded at 0.5 − 1 × 10^5^ cells/cm^2^. After passaging once by trypsinization, cryostocks (2 × 10^6^ cells/vial) were prepared upon 80% confluence (passage 2). Well differentiated PBEC cultures at ALI were generated by culturing passage 4 cells on 6.5 or 12 mm transwell inserts (Costar 3460) for 28 days in 1:1 AEGM/DMEM (Gibco) supplemented with 0.1 ng/mL retinoic acid as described previously^37^. Barrier formation was monitored by measuring electrical resistance with an EVOM2 and STX2 chopstick electrodes. During measurements, the basolateral compartment was filled with AEGM:DMEM, and the apical compartment with Phosphate-Buffered Saline. To calculate transepithelial electrical resistance (TEER) in Ω*cm^2^, the resistance of a blank transwell was subtracted from the resistance of the cell layer, followed by multiplication with the transwell surface area. Characteristics of the COPD and non-COPD donors are summarized in Table S1.

### RNA isolation and synthesis of cDNA

#### Human peripheral lung tissue

Total RNA was isolated from crushed human lung tissue using the Qiagen RNeasy kit (Qiagen, USA) according to the manufacturer’s instructions. Following analysis of the quantity and quality of the total RNA by nanodrop, total RNA (400 ng) was reverse transcribed into cDNA using the Transcriptor First Strand cDNA Synthesis Kit (Roche, Switzerland) following the instructions, including a no reverse transcription control and a no template control. Quantity and quality of the total RNA was verified by nanodrop analysis. Subsequently, cDNA was diluted in MilliQ (1:25) and stored in – 20 °C until use.

#### Undifferentiated PBEC

Total RNA was extracted from the undifferentiated PBEC (triplicates/donor) following the manufacturer’s protocol of the *mir*Vana^™^ kit (Thermo Fisher Scientific, USA/NL). Following analyzing the quantity and quality of the total RNA by nanodrop, total RNA (800 ng) was reverse transcribed into cDNA using the Transcriptor First Strand cDNA Synthesis Kit (Roche) following the instructions. Subsequently, cDNA was diluted in MilliQ (1:25) and stored in – 20 °C until use.

#### Differentiated PBEC

Differentiated PBEC (duplicates/donor) were lysed in TRIzol™ Reagent (Invitrogen^™^) at day 0 (day of air-lift; undifferentiated) and day 28 (28 days after air-lift; fully differentiated). Total RNA was extracted according to the manufacturer’s instructions (Catalog number 15596026 and 15596018, Invitrogen^™^) using glycogen blue co-precipitant (Thermo Fisher Scientific, USA). Nanodrop analysis of the RNA was conducted to verify quantity and quality of the total RNA. Next, cDNA synthesis was conducted from 350 ng total RNA using iScript^™^ cDNA synthesis kit (Bio-Rad, the Netherlands) including a no reverse transcription control and a no template control. cDNA dilutions were made in MilliQ (1:50) and stored at − 20 °C until use.

### Real time quantitative PCR analysis

Real-time quantitative PCR amplification was conducted by pipetting 4.4 µL of cDNA (diluted 1:25 or 1:50 in MilliQ water), 5 µL 2xSensiMix^™^ SYBR^®^ and Fluorescein Kit (Bioline, the Netherlands) and 0.6 µL forward and reverse primers for target genes in white LightCycler480 384 multiwell plates (Roche). Next, to study gene expression, these samples were analysed on the LightCycler480 machine (Roche) following the thermal cycling protocol: 10 min at 95 °C, 55 cycles of 10 s at 95 °C, 20 s at 60 °C. The list of target-specific primers used for qPCR analysis is shown in Table S2.

Qualitative analysis of melt curves and peaks using LightCycler480 software (Roche) was used to check for a-specific amplification which were subsequently excluded. Quantitative analysis (i.e., N-zero values: extrapolated relative amount of DNA at cycle zero) by LinRegPCR software 2014.x (the Netherlands) was used to calculate gene expression. Exclusion of samples with no amplification, no plateau phase, too low Cq value or outside 5% of group mean was automatically conducted during LinRegPCR analysis. GeNorm software 3.4 (Primerdesign, USA) was used to calculate the geometric mean of a combination of at least two and up to four housekeeping genes (*Actin B* (*ACTB*), *Beta-2 Microglobulin* (*B2M*), *Peptidylprolyl Isomerase A* (*PPIA*), *Ribosomal Protein L13A* (*RPL13A*)), which was used for normalization of the calculated expression of target genes.

### Western blotting

#### Human peripheral lung tissue

Crushed human lung tissue was lysed in buffer containing 50 mM Tris pH 7.4, 150 mM NaCl, 0.1% NP40 and protease inhibitor cocktail (Roche).

#### Undifferentiated PBEC

Undifferentiated PBEC (quintuplicates/donor) were lysed in RIPA buffer (50 mM Tris pH 8.0, 150 mM NaCl, 1% Nonidet P40 or 1% Triton X-100, 0.5% NA deoxycholate, 0.1% sodium dodecyl sulphate in MilliQ) including fresh non-stable components: PIC, 1 mM sodium orthovanadate.

#### Differentiated PBEC

Differentiated PBEC (duplicates/donor) were lysed at day 28 in Whole Cell Lysis buffer (20 mM Tris pH 7.4, 150 mM NaCl, 1% Nonidet P40 in MilliQ) including PhosSTOP Phosphatase and cOmplete, Mini, EDTA-free Protease Inhibitor cocktail tablets (both Roche).

Whole cell lysates of human peripheral lung tissue, undifferentiated and differentiated PBEC experiments were rotated for 30 min at 4 °C and centrifuged at 20,000 × g for 30 min at 4 °C. Thereafter, the total protein content was analysed by the Pierce^™^ BCA Protein Assay Kit (Catalog number 23225 and 23227, Thermo Fisher Scientific), and whole cell lysates were diluted in a final concentration of 0.19–0.71 µg/µL in 1 × Laemmli buffer (0.25 M Tris pH 6.8, 8% (w/v) sodium dodecyl sulphate, 40% (v/v) glycerol, 0.4 M Dithiothreitol, 0.02% (w/v) Bromophenol Blue). These diluted lysates were boiled for 5 min at 100 °C and subsequently stored at − 20 °C (undifferentiated PBEC) or − 80 °C (lung homogenates/differentiated PBEC) until further analysis. The protein ladders (Precision Plus Protein™ All Blue Standards #161-0373, Bio-Rad) and protein samples were loaded on a Criterion XT Precast 4–12% or 12% Bis–Tris gel (Bio-Rad), respectively singular peripheral lung homogenates per donor (7.14 µg/sample), pool of quintuplicates per undifferentiated PBEC donor (10 µg/sample), and duplicates per differentiated PBEC donor (2.75 µg/sample). Proteins were separated in 1 × MES running buffer (Bio-Rad) by electrophoresis (100–130 V for 1 h), whereafter proteins were transferred from the gel to a 0.45 µM Nitrocellulose Transfer membrane (Bio-Rad) by electroblotting (100 V for 1 h) using a Bio-Rad Criterion Blotter. To stain and correct for total protein content, the nitrocellulose membranes were incubated with Ponceau S staining (0.2% in 1% acetic acid; Sigma-Aldrich) which was visualized using the Amersham™ Imager 600 (GE Healthcare, the Netherlands). To stain the target-specific proteins, the Ponceau S staining was washed off and the membranes were blocked for 1 h in 3% (w/v) non-fat dry milk (Campina, the Netherlands) dissolved in Tween20 Tris-buffered saline (TBST; 20 mM Tris, 137 mM NaCl, 0.1% (v/v) Tween20, pH 7.6). After finishing blocking, the membranes were washed with TBST and incubated with a target-specific primary antibody diluted in 3% (w/v) BSA at 4 °C overnight. Next, the membranes were again washed with TBST and now incubated with a horseradish peroxidase-conjugated secondary antibody diluted in 3% (w/v) non-fat dry milk in TBST for 1 h at room temperature. To visualize the target proteins, the membranes were washed with TBST, stained with 0.25-1 × Supersignal West FEMTO and 0.5-1 × Supersignal West PICO Chemiluminescent Substrate (Thermo Fisher Scientific), and imaged using the Amersham^™^ Imager 600. The list of primary and secondary antibodies used for western blotting is shown in Table S3. The Image Quant software (GE Healthcare) was used for quantification of the total protein content and target-specific proteins. Total protein content was quantified using the Ponceau S-stained images over the entire size range of proteins (250 kDa-10 kDa). This total protein content was used as normalization factor for (quantification of) target-specific proteins. Because several proteins of interest were detected on a similar gel, which is possible due to their difference in molecular mass, correction for total protein content/loading of these target proteins was based on the same Ponceau S staining. To specify, Gel I: HK2, DNM1L, OXPHOS, SQSTM1, TOMM20; Gel II: PRKN, BNIP3L, BNIP3, FUNDC1, PINK1, MAP1LC3B; Gel III: NRF1, TFAM, GABARAPL1, ESRRA.

### Image acquisition: equipment and settings

We performed chemiluminescence capturing with automatic or manual exposure time as described in the Operating Instructions of the Amersham^™^ Imager 600 (GE Healthcare). A short pre-exposure is performed to determine the signal intensity. The system will use this information to calculate which exposure time will give the highest possible signal below saturation to enable accurate quantification of the sample.’ Manual exposure is only performed with a maximum duration of 5 min, in case the intensity of the image is inadequate (i.e., takes > 5–10 min) after automatic exposure, which is also suggested by the manufacturer’ instructions. Western blot images displayed in the (supplementary) figures of this manuscript have been adjusted for brightness and contrast equally throughout the picture. In addition, the Ponceau S band (37, 50, 70 or 75 kDa) presented in the (supplementary) figures is representative for the total Ponceau S staining per sample. For illustration purposes of the protein abundance in the human peripheral lung tissue homogenates in the manuscript figures, examples of bands of the target proteins along with the corresponding normalization bands of the Ponceau S staining are shown of one patient/group, which is not always representative for the mean and changes in the whole patient group as quantified in the box plots (due to the variation between patients). In the Supplementary Figs.1–3, all western blot images of the target proteins along with the corresponding normalization bands of the Ponceau S staining of all subjects/group are shown of the peripheral lung tissue from non-COPD patients and COPD patients (GOLDII; GOLDIV) (single) (Figure S1), as well as images of undifferentiated PBEC (pool of quintuplicates) (Figure S2) and differentiated PBEC (duplicates) (Figure S3) from non-COPD patients and COPD patients.

### Statistical data analysis

GraphPad Prism 8.0 software (La Jolla, USA) was used to display the data in box plot or scatter plot graphs. Data are presented as mean fold change of COPD patients (GOLDII or GOLDIV) relative to non-COPD patients (box plot: min to max; scatter plot: ± standard error of the mean (SEM)). Individual subjects are represented by open circles (non-COPD patients), squares (all COPD patients), triangles (COPD GOLDII patients) and diamonds (COPD GOLDIV patients). Quality of the mRNA data was analysed by melt curve/peak analysis using LightCycler480 software (Roche). In addition, exclusion of extreme outliers in the analysis of peripheral lung tissue was also based on box plot analysis (i.e., values more than three interquartile ranges from the end of a box) in IBM Statistics SPSS 25. Statistical differences were analysed using GraphPad Prism 8.0 software. First, normal distribution of the data was assessed with the Shapiro–Wilk test. Secondly, in case of testing the difference between two groups (COPD patients *versus* non-COPD patients), a two-tailed unpaired parametric t-test (normal distribution) or non-parametric Mann–Whitney test (non-normal distribution) was used. In addition, in case of testing the difference between three groups (COPD GOLDII patients or COPD GOLDIV patients relative to non-COPD patients), we used a one-way ANOVA and a Dunnett’s post-hoc test for multiple comparisons (normal distribution) or a Kruskal–Wallis test followed by a Dunn’s multiple comparisons test (non-normal distribution). Moreover, the correlation between lung function (forced expiratory volume in first second/forced vital capacity; FEV_1_/FVC) and transcript levels of investigated markers was evaluated by calculating the Spearman’s correlation coefficient (ρ) using IBM SPSS Statistics version 25.0.

With respect to the validation of the PBEC differentiation, TEER and gene expression data are presented as mean fold change of day 0 *versus* day 28 per patient group. Statistical differences between day 28 (differentiated) relative to day 0 (undifferentiated) in separate patient groups were tested using a two-tailed paired parametric t-test, or in case of non-normal distribution a paired nonparametric Wilcoxon test. Moreover, differences between day 28 (differentiated) of non-COPD relative to COPD patients were tested using a two-tailed unpaired parametric t-test (normal distribution) or non-parametric Mann–Whitney test (non-normal distribution). Statistical significance was considered if p-values were below 0.05 (^*^p < 0.05) or 0.01 (^**^p < 0.01) and a trend was indicated if below 0.1 (^#^p < 0.1).

### Ethics approval and consent to participate

All methods were performed in accordance with the relevant guidelines and regulations. Human peripheral lung tissue: The study was approved by the medical ethical committee of the MUMC + , Maastricht, the Netherlands (MEC 02-043.3). All subjects gave their informed consent in writing. Undifferentiated PBEC: The scientific board of the MPTC approved the use of materials for this study under MPTC2010-019. In addition, the local medical ethical committee of the MUMC+ , Maastricht, the Netherlands approved the use of human tissue (METC 2017-0087). Differentiated PBEC: The study was approved by the local ethical committee Ethik-Kommission des Fachbereichs Humanmedizin der Philipps-Universität Marburg, Marburg, Germany (Marburg 223/12).

## Results

### Characteristics of COPD patients and non-COPD patients

Peripheral lung tissue was retrieved from non-COPD patients and COPD patients, of which the characteristics are described in Table 1. Obviously, lung function was significantly lower in the group of COPD patients (FEV_1_/FVC: 39.0 ± 5.1) *versus* non-COPD patients (FEV_1_/FVC: 83.1 ± 2.8). When COPD patients were categorized according to disease status (moderate: GOLDII and very severe: GOLDIV), the group of moderate COPD patients included only current-smoking males of an older age (69.8 ± 4.2) while the very severe COPD disease group consisted of mostly ex-smoking females (one ex-smoking male) of a younger age (53.3 ± 3.0). The differences in smoking status between these groups can be explained by the reason for surgery. Non-COPD patients and COPD GOLDII patients underwent surgery to remove a lung tumor and COPD GOLDIV patients underwent lung volume reduction surgery which requires smoking cessation. Body Mass Index (BMI) and pack-years were equally distributed among the groups.

Characteristics of non-COPD patients and COPD patients of which PBECs were cultured submerged (undifferentiated) or at ALI (differentiated) are summarized in Table S1. As expected, lung function was significantly lower in the group of COPD patients in both undifferentiated PBEC (FEV_1_/FVC: COPD: 60.2 ± 2.3 *versus* non-COPD: 79.3 ± 3.4) and differentiated PBEC (FEV_1_/FVC: COPD: 50.8 ± 4.0 *versus* non-COPD: 77.3 ± 2.0). In general, gender, age and BMI were equally distributed among the COPD and non-COPD patients in both PBEC studies. The only exception, COPD patients in the undifferentiated PBEC and both patient groups in the differentiated PBEC study included only males. Remarkably, pack-years was significantly higher in the group of COPD patients in differentiated PBEC (43.8 ± 5.7) *versus* non-COPD patients (7.5 ± 4.0).

### Expression of epithelial cell markers in differentiated PBEC from COPD patients and non-COPD patients

Validation and characterization of the differentiation of PBEC was conducted by TEER (as an indication of the integrity of the formation of the epithelial barrier during differentiation) and by gene expression analysis of cell type-specific markers of basal and luminal epithelial cells on day 0 (undifferentiated) *versus* day 28 (fully differentiated).

As depicted in Figure S4A, differentiated PBEC from both COPD patients and non-COPD patients showed a significant increase in TEER from day 0 to day 28, while no differences were shown in TEER at day 28 between COPD and non-COPD patients. Moreover, as shown in Figures S4B and S4C, we observed a significant decrease in mRNA levels of basal epithelial cell markers (*Tumor Protein P63* (*TP63*), *Keratin*-5 (*KRT5*) and *Keratin-14* (*KRT14*)) as well as a profound increased expression of club cell secretory marker (*Secretoglobin Familiy 1A Member 1* (*SCGB1A1*)), ciliogenesis marker (*Forkhead Box J1* (*FOXJ1*)) and goblet cell differentiation marker (*Mucin 5AC, Oligomeric Mucus/Gel-Forming* (*MUC5AC*)) at day 28 compared to day 0 of differentiation in both non-COPD patients and COPD patients. Lastly, in COPD patients *versus* non-COPD patients, we observed that transcript levels of genes associated with club cell differentiation and ciliogenesis were decreased, while increased levels of a marker of goblet cell differentiation were observed at day 28 (Figure S4B,C).

### Downregulation of key constituents controlling mitochondrial biogenesis in peripheral lung tissue from very severe COPD patients

To evaluate if, and to what extent, the pathways regulating mitochondrial turnover (biogenesis *versus* breakdown of mitochondria) are affected in peripheral lung tissue from COPD patients, we first investigated the mRNA expression of molecules involved in the regulation of mitochondrial biogenesis.

**Figure 1:**
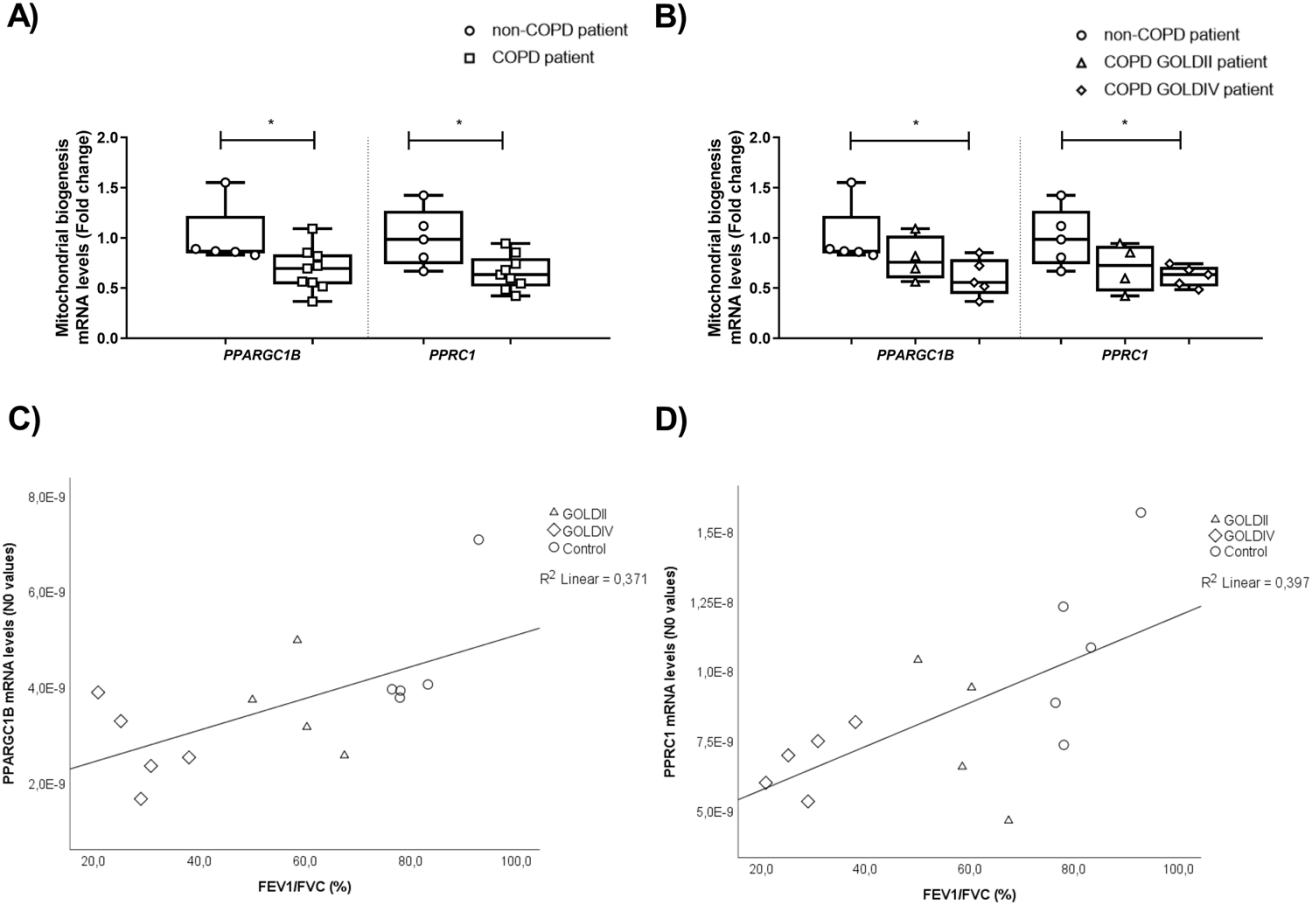
Decreased abundance of molecules controlling mitochondrial biogenesis in peripheral lung tissue from severe COPD patients. Real-time qPCR analysis was used to analyse mRNA expression of *PPARGC1B* and *PPRC1* in human peripheral lung tissue from non-COPD patients (n = 5) and COPD patients (n = 9; GOLDII n = 4/9, GOLDIV n = 5/9) (**A**, **B**). Data are presented as mean fold change of COPD patients (GOLDII or GOLDIV) relative to non-COPD patients in box plots (min to max). Statistical differences between COPD patients *versus* non-COPD patients (two groups) were tested using a two-tailed unpaired parametric t-test (normal distribution), or an unpaired nonparametric Mann–Whitney test (non-normal distribution). In addition, in case of testing the difference between COPD GOLDII or GOLDIV patients *versus* non-COPD patients (three groups), we used a one-way ANOVA and a Dunnett’s post-hoc test for multiple comparisons (normal distribution) or a Kruskal–Wallis test followed by a Dunn’s multiple comparisons test (non-normal distribution). Moreover, the Spearman’s correlation coefficient assessed the correlation between lung function (FEV_1_/FVC) and transcript levels of *PPARGC1B* and *PPRC1* in human peripheral lung tissue (n = 14) (**C**, **D**). Individual subjects are represented by open circles (non-COPD patients), squares (all COPD patients), triangles (COPD GOLDII patients) and diamonds (COPD GOLDIV patients). Statistical significance is indicated as ^*^p < 0.05 compared to non-COPD patients.

Peripheral lung tissue from COPD patients showed decreased mRNA levels of *PPARGC1B* and *PPRC1* compared to non-COPD patients (Fig. 1A). Interestingly, upon further inspection, a significant downregulation of *PPARGC1B* and *PPRC1* transcript levels was shown in COPD GOLDIV patients compared to non-COPD patients (Fig. 1B). Moreover, a significant positive correlation between lung function, i.e., percentage FEV_1_/FVC, and *PPARGC1B* expression (ρ = 0.609, p = 0.021; R^2^ = 0.371) as well as between lung function and *PPRC1* expression (ρ = 0.613, p = 0.020; R^2^ = 0.397) was observed (Figs. 1C,D).

**Figure 2:**
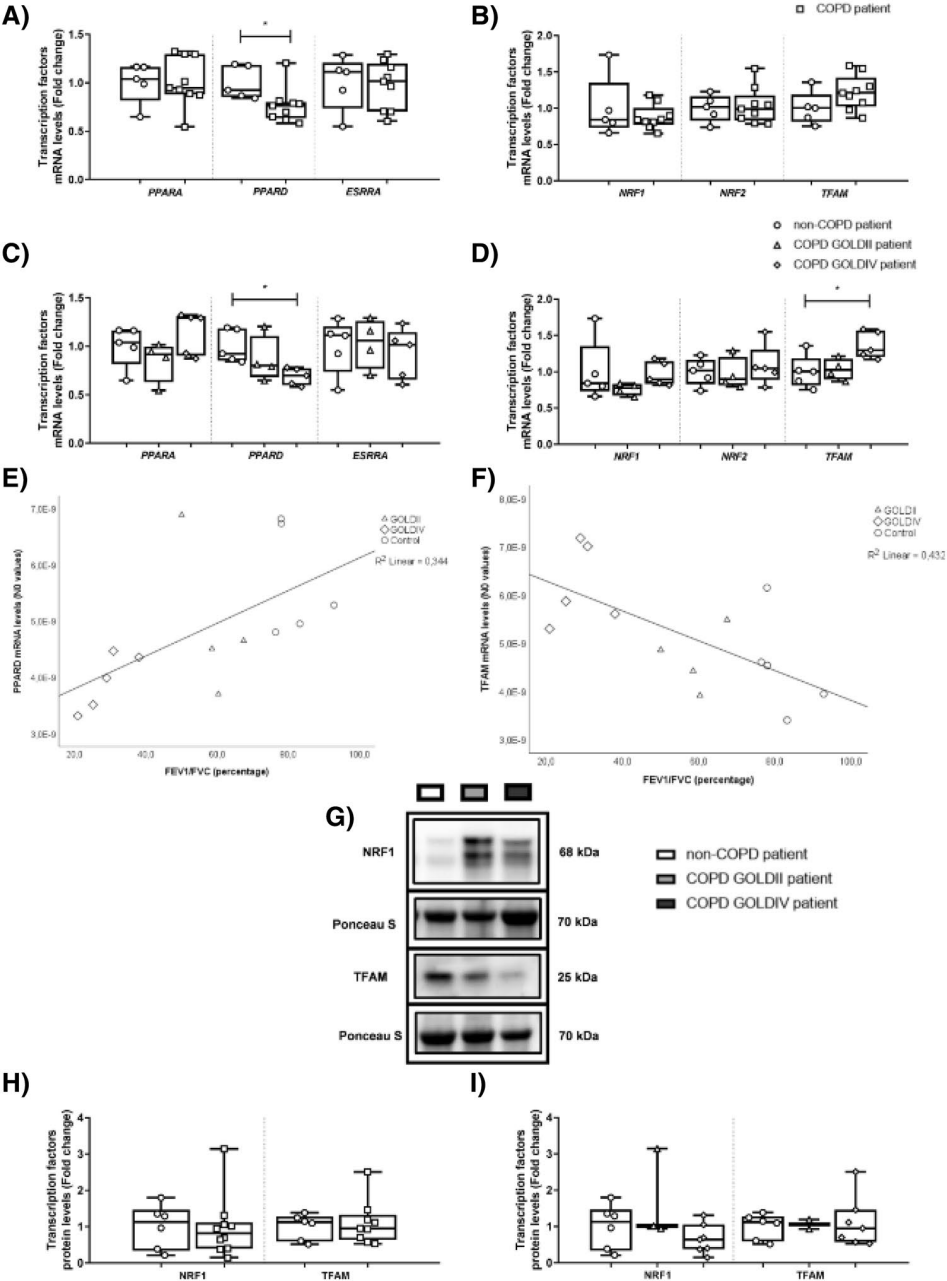
Altered expression of PPARGC1-co-activated transcription factors in peripheral lung tissue from very severe COPD patients. Real-time qPCR analysis was used to analyse mRNA expression of *PPARA*, *PPARD* and *ESRRA* **(A, C)** as well as *NRF1*, *NRF2*, and *TFAM* (**B**, **D**) in human peripheral lung tissue from non-COPD patients (n = 5) and COPD patients (n = 9; GOLDII n = 4/9, GOLDIV n = 5/9). Furthermore, Spearman’s correlation coefficient has been determined to evaluate the correlation between lung function (FEV_1_/FVC) and transcript levels of *PPARD* or *TFAM* (**E**, **F**) in human peripheral lung tissue (n = 14). Protein levels of NRF1 and TFAM were assessed in human peripheral lung tissue from non-COPD patients (n = 6) and COPD patients (n = 9–10; GOLDII n = 2–3/9–10, GOLDIV n = 7/9–10) by western blot analysis (**G-I**). For illustration purposes of the protein abundance in the human peripheral lung tissue homogenates, examples of bands of the target proteins along with the corresponding normalization bands of the Ponceau S staining are shown of one patient/group, which is not always representative for the mean and changes in the whole patient group as quantified in the box plots (due to the variation between patients). Black boxes represent a series of bands for specific proteins that were cut from the same blot. Data are presented as mean fold change of COPD patients (GOLDII or GOLDIV) relative to non-COPD patients in box plots (min to max). Statistical differences between COPD patients *versus* non-COPD patients (two groups) were tested using a two-tailed unpaired parametric t-test (normal distribution) or an unpaired nonparametric Mann–Whitney test (non-normal distribution). In addition, in case of testing the difference between COPD GOLDII or GOLDIV patients *versus* non-COPD patients (three groups), we used a one-way ANOVA and a Dunnett’s post-hoc test for multiple comparisons (normal distribution) or a Kruskal–Wallis test followed by a Dunn’s multiple comparisons test (non-normal distribution). Individual subjects are represented by open circles (non-COPD patients), squares (all COPD patients), triangles (COPD GOLDII patients) and diamonds (COPD GOLDIV patients). Statistical significance is indicated as ^*^p < 0.05 compared to non-COPD patients.

Next, we explored the abundance of PPARGC1-coactivated transcription factors in peripheral lung tissue from COPD and non-COPD patients. Although the transcript levels of most transcription factors that we analysed were not different between COPD and non-COPD patients, we observed a significant decrease in mRNA expression of PPAR delta (*PPARD*) in COPD patients (Fig. 2A,B). This decline in *PPARD* abundance was in particular observed in peripheral lung tissue from COPD GOLDIV patients relative to non-COPD patients (Fig. 2C). In addition, stratification of the findings according to disease status revealed an increase in the expression of *TFAM* in COPD GOLDIV patients compared to non-COPD patients (Fig. 2D). We next assessed whether these differences in transcript levels of *PPARD* and *TFAM* correlated with lung function parameters. Indeed, correlation analyses revealed a positive correlation between lung function parameters and transcript levels of *PPARD* (ρ = 0.741, p = 0.002; R^2^ = 0.344), while a negative correlation was observed between lung function and *TFAM* expression (ρ = − 0.613, p = 0.020; R^2^ = 0.432) (Figs. 2E,F). Lastly, we assessed the protein abundance of transcription factors involved in mitochondrial biogenesis and energy metabolism, NRF1 and TFAM, but no significant alterations were observed between COPD and non-COPD patients (Fig. 2G–I) (Figure S1).

In summary, this data shows that, with the exception of an upregulation of *TFAM* mRNA expression, transcript abundance of molecules involved in the regulation of mitochondrial biogenesis by the PPARGC1 family is downregulated in peripheral lung tissue from COPD patients, in particular in COPD GOLDIV patients.

In line with the alterations in peripheral lung tissue as described above, we did observe decreased transcript levels of *PPARGC1B* between COPD and non-COPD patients in undifferentiated as well as differentiated PBEC cultures. No pronounced changes in the abundance of other molecules controlling mitochondrial biogenesis were observed in both PBEC models (Tables S4 and S5; Figures S2 and S3).

### Increased transcript levels of BNIP3L in peripheral lung tissue from COPD patients

Besides mitochondrial biogenesis, mitochondrial quality control by the selective breakdown of damaged or defected mitochondria (i.e., mitophagy) also plays a critical role in maintaining mitochondrial homeostasis. Therefore, we next assessed the protein and mRNA abundance of key molecules involved in both receptor-mediated and ubiquitin-mediated mitophagy in peripheral lung tissue and PBEC from COPD patients.

**Figure 3:**
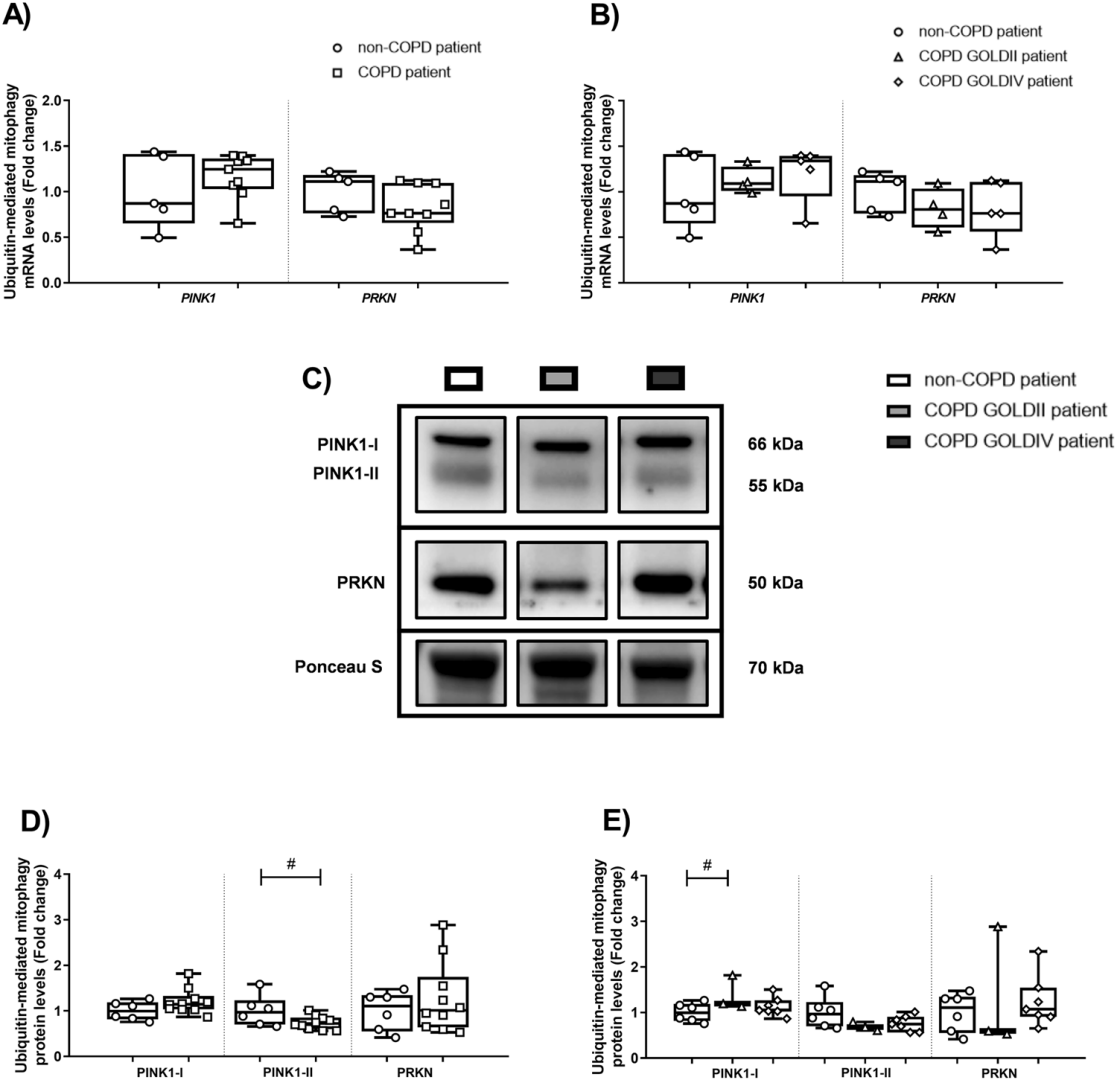
The abundance of constituents involved in ubiquitin-mediated mitophagy in peripheral lung tissue from COPD patients. Real-time qPCR analysis was used to analyse mRNA expression of *PINK1* and *PRKN* in human peripheral lung tissue from non-COPD patients (n = 5) and COPD patients (n = 9; GOLDII n = 4/9, GOLDIV n = 5/9) (**A**, **B**). Protein levels of PINK1-I, PINK1-II and PRKN were assessed in human peripheral lung tissue from non-COPD patients (n = 6) and COPD patients (n = 10; GOLDII n = 3/10, GOLDIV n = 7/10) by western blot analysis (**C**-**E**). Western blot analysis revealed two distinct bands corresponding with expected molecular weights for PINK1 protein, entitled PINK1-I (66 kDa) and PINK1-II (55 kDa). For illustration purposes of the protein abundance in the human peripheral lung tissue homogenates, examples of bands of the target proteins along with the corresponding normalization bands of the Ponceau S staining are shown of one patient/group, which is not always representative for the mean and changes in the whole patient group as quantified in the box plots (due to the variation between patients). The smaller black boxes represent bands of specific proteins from individuals that were cut from the same blot. Data are presented as mean fold change of COPD patients (GOLDII or GOLDIV) relative to non-COPD patients in box plots (min to max). Statistical differences between COPD patients *versus* non-COPD patients (two groups) were tested using a two-tailed unpaired parametric t-test (normal distribution). In addition, in case of testing the difference between COPD GOLDII or GOLDIV patients *versus* non-COPD patients (three groups), we used a one-way ANOVA and a Dunnett’s post-hoc test for multiple comparisons (normal distribution) or a Kruskal–Wallis test followed by a Dunn’s multiple comparisons test (non-normal distribution). Individual subjects are represented by open circles (non-COPD patients), squares (all COPD patients), triangles (COPD GOLDII patients) and diamonds (COPD GOLDIV patients). A trend is indicated as ^#^p < 0.1 compared to non-COPD patients.

**Figure 4:**
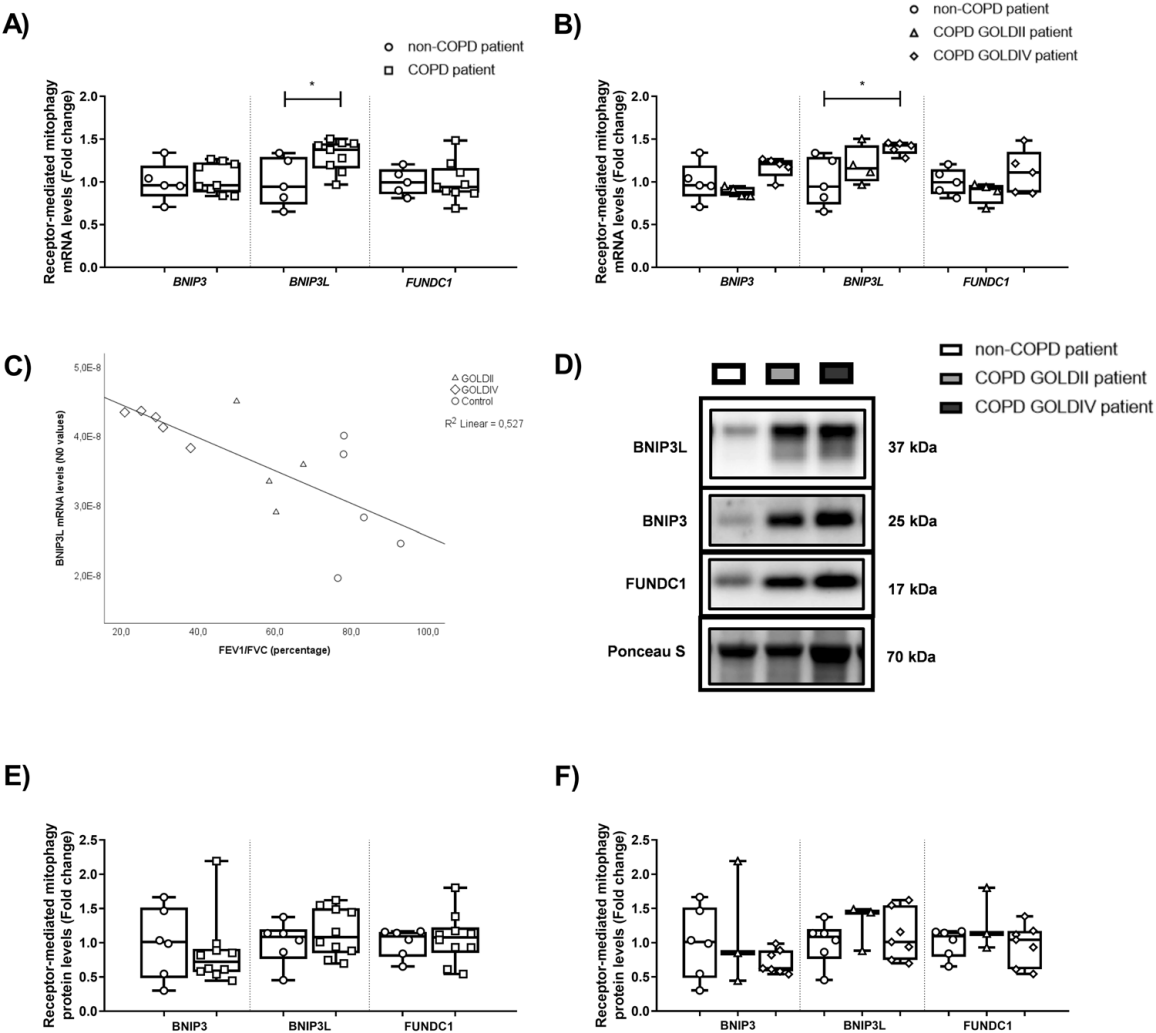
Increased transcript levels of mitophagy protein *BNIP3L* in peripheral lung tissue from very severe COPD patients. Real-time qPCR analysis was used to analyse mRNA expression of *BNIP3*, *BNIP3L* and *FUNDC1* in human peripheral lung tissue from non-COPD patients (n = 5) and COPD patients (n = 9; GOLDII n = 4/9, GOLDIV n = 5/9) (**A**, **B**). Moreover, the Spearman’s correlation coefficient has been determined to evaluate the correlation between lung function (FEV_1_/FVC) and transcript levels of *BNIP3L* in human peripheral lung tissue (n = 14) (**C**). Protein levels of BNIP3, BNIP3L, and FUNDC1 were assessed in human peripheral lung tissue from non-COPD patients (n = 6) and COPD patients (n = 10; GOLDII n = 3/10, GOLDIV n = 7/10) by western blot analysis (**D–F**). For illustration purposes of the protein abundance in the human peripheral lung tissue homogenates, examples of bands of the target proteins along with the corresponding normalization bands of the Ponceau S staining are shown of one patient/group, which is not always representative for the mean and changes in the whole patient group as quantified in the box plots (due to the variation between patients). Black boxes represent a series of bands for specific proteins that were cut from the same blot. Data are presented as mean fold change of COPD patients (GOLDII or GOLDIV) relative to non-COPD patients in box plots (min to max). Statistical differences between COPD patients *versus* non-COPD patients (two groups) were tested using a two-tailed unpaired parametric t-test (normal distribution), or an unpaired nonparametric Mann–Whitney test (non-normal distribution). In addition, in case of testing the difference between COPD GOLDII or GOLDIV patients *versus* non-COPD patients (three groups), we used a one-way ANOVA and a Dunnett’s post-hoc test for multiple comparisons (normal distribution). Individual subjects are represented by open circles (non-COPD patients), squares (all COPD patients), triangles (COPD GOLDII patients) and diamonds (COPD GOLDIV patients). Statistical significance is indicated as ^*^p < 0.05 compared to non-COPD patients.

No pronounced differences in mRNA and protein levels of key regulators associated with ubiquitin-mediated mitophagy were observed in peripheral lung tissue from COPD patients (Fig. 3A–E; Figure S1). Also, there was no correlation between lung function parameters and genes associated with ubiquitin-mediated mitophagy (data not shown). Interestingly, with respect to receptor-mediated mitophagy, significantly increased transcript levels of *BNIP3L* were observed in peripheral lung tissue from COPD patients, in particular in very severe COPD patients (Figs. 4A,B). This increased expression of *BNIP3L* negatively correlated with lung function (ρ = − 0.745, p = 0.002; R^2^ = 0.527) (Fig. 4C). In contrast to transcript levels, no changes were observed in the expression of proteins involved in receptor-mediated mitophagy in lung tissue from COPD patients relative to non-COPD patients (Figs. 4D,F; Figure S1).

As autophagy is required to facilitate the degradation of mitochondria by formation of an autophagosome, we investigated if the expression of specific autophagy proteins was affected in lung tissue from COPD patients. In summary, we observed no alterations in either protein nor gene expression of molecules involved in autophagy in peripheral lung tissue from COPD patients relative to non-COPD patients (Table 2; Figure S1).

**Table 2:** Abundance of molecules involved in autophagy in peripheral lung tissue from COPD patients.

Level of expression	Marker	Non-COPD patients (n = 5–6)	COPD patients (n = 9–10)	GOLDII patients (n = 3–4)	GOLDIV patients (n = 5–7)	p-value COPD vs non-COPD
Autophagy						
Protein	SQSTM1	1.0 ± 0.09	1.1 ± 0.14	1.4 ± 0.40	1.0 ± 0.11	0.48
	GABARAPL1	1.0 ± 0.06	0.93 ± 0.08	1.1 ± 0.15	0.86 ± 0.09	0.55
	Ratio MAP1LC3BII/I	1.0 ± 0.10	0.99 ± 0.07	0.77 ± 0.05	1.1 ± 0.07	0.93
mRNA	*SQSTM1*	1.0 ± 0.15	0.95 ± 0.03	0.94 ± 0.04	0.96 ± 0.05	0.69
	*GABARAPL1*	1.0 ± 0.16	0.97 ± 0.07	0.89 ± 0.08	1.0 ± 0.10	0.85
	*MAP1LC3A*	1.0 ± 0.06	0.82 ± 0.13	0.83 ± 0.22	0.80 ± 0.19	0.35
	*MAP1LC3B*	1.0 ± 0.11	1.0 ± 0.05	1.0 ± 0.09	0.97 ± 0.05	1.00

Protein levels of SQSTM1, GABARAPL1, ratio MAP1LC3BII/I were assessed in human peripheral lung tissue from non-COPD patients (n = 6) and COPD patients (n = 10; GOLDII n = 3/10, GOLDIV n = 7/10) by western blot analysis. In addition, mRNA expression of *SQSTM1*, *GABARAPL1*, *MAP1LC3A* and *MAP1LC3B* was analysed in human peripheral lung tissue from non-COPD patients (n = 5) and COPD patients (n = 9; GOLDII n = 4/9, GOLDIV n = 5/9). Data are presented as mean fold change of COPD patients (GOLDII or GOLDIV) relative to non-COPD patients ± SEM. Statistical differences between COPD patients *versus* non-COPD patients (two groups) were tested using a two-tailed unpaired parametric t-test (normal distribution) (p-value shown in last column). In addition, in case of testing the difference between COPD GOLDII or GOLDIV patients *versus* non-COPD patients (three groups), we used a one-way ANOVA and a Dunnett’s post-hoc test for multiple comparisons (normal distribution) or a Kruskal–Wallis test followed by a Dunn’s multiple comparisons test (non-normal distribution) (p-value not shown).

Elevated mRNA levels of *BNIP3L, as depicted in *Fig. 4*,* were not observed in undifferentiated or differentiated PBEC from COPD patients. Moreover, in accordance with our other findings in peripheral lung tissue, the abundance of mitophagy and autophagy regulators was not different in PBECs from COPD-patients compared to non-COPD patients (Tables S4 and S5; Figs. S2 and S3).

With regard to mitophagy and autophagy, in summary, the expression of *BNIP3L* is elevated in peripheral lung tissue, not in PBEC, from very severe COPD patients , while other markers associated with mitophagy and autophagy were not differently-expressed in COPD patients relative to non-COPD patients.

### No pronounced changes in the expression of constituents of mitochondrial metabolic processes and mitochondrial dynamics in peripheral tissue or PBEC from COPD patients

Given the changes in expression of markers of mitochondrial biogenesis and mitophagy, we investigated transcript and protein abundance of a myriad of constituents of different mitochondrial metabolic processes including the electron transport chain, tricarboxylic acid cycle, glycolysis, and fatty acid β-oxidation. As shown in Table 3 and Figure S1, no pronounced differences in abundance of these regulators were observed in peripheral lung tissue from COPD patients *versus* non-COPD patients, except for minor changes in the transcript abundance of subunits of complex III (decrease) and complex IV (increase). Lastly, we explored the regulation of mitochondrial dynamics and observed unaltered expression of fission- and fusion-associated proteins in peripheral lung tissue from COPD patients, sustained during different disease stages (Table 4; Figure S1). In line with these results, we did not observe pronounced alterations in the regulation of mitochondrial content, metabolism or dynamics in undifferentiated or differentiated PBEC cultures from COPD-patients compared to non-COPD patients, except a downregulation of *OPA1* expression (mitochondrial fission) in differentiated PBEC from COPD patients (Tables S4 and S5; Figs. S2 and S3).

**Table 3:** Abundance of constituents involved in mitochondrial content/metabolism in peripheral lung tissue from COPD patients.

Level of expression	Marker	Non-COPD patients (n = 5–6)	COPD patients (n = 9–10)	GOLDII patients (n = 3–4)	GOLDIV patients (n = 5–7)	p-valueCOPD vs non-COPD
Electron transport chain						
Protein	NDUFB8 (COXI)	1.0 ± 0.12	1.1 ± 0.13	1.1 ± 0.15	1.0 ± 0.17	0.71
	SDHB (COXII)	1.0 ± 0.17	0.91 ± 0.07	1.0 ± 0.15	0.86 ± 0.08	0.59
	UQCRC2 (COXIII)	1.0 ± 0.26	1.9 ± 0.76	3.6 ± 2.4	1.2 ± 0.39	0.56
	ATP5A (COXV)	1.0 ± 0.11	1.1 ± 0.10	1.2 ± 0.13	0.96 ± 0.13	0.78
mRNA	*NDUFB3 (COXI)*	1.0 ± 0.06	1.0 ± 0.06	1.1 ± 0.13	1.0 ± 0.05	0.61
	*CYC1 (COXIII)*	1.0 ± 0.06	1.0 ± 0.05	1.0 ± 0.02	0.99 ± 0.08	0.89
	*CYTB (COXIII)*	1.0 ± 0.14	0.64 ± 0.09	0.76 ± 0.05	0.54 ± 0.14*	0.04*
	*COXII (COXIV)*	1.0 ± 0.14	0.79 ± 0.06	0.75 ± 0.09	0.82 ± 0.08	0.30
	*COXIII (COXIV)*	1.0 ± 0.16	0.77 ± 0.07	0.71 ± 0.07	0.82 ± 0.11	0.30
	*COXIV (COXIV)*	1.0 ± 0.05	1.2 ± 0.03	1.2 ± 0.05^#^	1.2 ± 0.05^#^	0.02*
Translocase of outer membrane						
Protein	TOMM20	1.0 ± 0.12	0.99 ± 0.15	1.5 ± 0.34	0.79 ± 0.10	0.96
Tricarboxylic acid cycle						
mRNA	*Citrate synthase*	1.0 ± 0.09	1.1 ± 0.05	1.1 ± 0.08	1.1 ± 0.08	0.24
	*PDK4*	1.0 ± 0.27	0.77 ± 0.10	0.88 ± 0.18	0.69 ± 0.13	0.36
Glycolysis						
Protein	HK2	1.0 ± 0.22	1.3 ± 0.16	1.8 ± 0.09^#^	1.1 ± 0.17	0.30
mRNA	*HK2*	1.0 ± 0.16	1.3 ± 0.13	1.5 ± 0.19	1.1 ± 0.17	0.23
Fatty acid β-oxidation						
mRNA	*HADH*	1.0 ± 0.06	1.0 ± 0.05	1.1 ± 0.08	1.0 ± 0.08	0.44

Protein levels of subunits of electron transport chain complexes, TOMM20, and molecules involved in glycolysis were assessed in human peripheral lung tissue from non-COPD patients (n = 6) and COPD patients (n = 10; GOLDII n = 3/10, GOLDIV n = 7/10) by western blot analysis. In addition, mRNA expression of subunits of electron transport chain complexes, and regulators involved in the tricarboxylic acid cycle, glycolysis and fatty acid β–oxidation was analysed in human peripheral lung tissue from non-COPD patients (n = 5) and COPD patients (n = 9; GOLDII n = 4/9, GOLDIV n = 5/9). Data are presented as mean fold change of COPD patients (GOLDII or GOLDIV) relative to non-COPD patients ± SEM. Statistical differences between COPD patients *versus* non-COPD patients (two groups) were tested using a two-tailed unpaired parametric t-test (normal distribution), or an unpaired nonparametric Mann–Whitney test (non-normal distribution) (p-value shown in last column). In addition, in case of testing the difference between COPD GOLDII or GOLDIV patients *versus* non-COPD patients (three groups), we used a one-way ANOVA and a Dunnett’s post-hoc test for multiple comparisons (normal distribution) or a Kruskal–Wallis test followed by a Dunn’s multiple comparisons test (non-normal distribution) (p-value not shown). Statistical significance is indicated as *p < 0.05 and a trend was indicated if ^#^p < 0.1 compared to non-COPD patients.

**Table 4:** Expression of fission- and fusion-associated proteins in peripheral lung tissue from COPD patients.

Level of expression	Marker	Non-COPD patients (n = 5–6)	COPD patients (n = 9–10)	GOLDII patients (n = 3–4)	GOLDIV patients (n = 5–7)	p-value COPD vs non-COPD
Fission						
Protein	DNM1L	1.0 ± 0.12	1.0 ± 0.11	1.3 ± 0.11	0.93 ± 0.13	0.86
mRNA	*DNM1L*	1.0 ± 0.08	0.99 ± 0.05	0.95 ± 0.07	1.0 ± 0.07	0.87
	*FIS1*	1.0 ± 0.13	1.2 ± 0.06	1.1 ± 0.03	1.3 ± 0.09	0.16
Fusion						
mRNA	*MFN1*	1.0 ± 0.13	0.94 ± 0.05	0.91 ± 0.09	0.96 ± 0.05	> 0.99
	*MFN2*	1.0 ± 0.13	0.96 ± 0.03	0.97 ± 0.06	0.96 ± 0.05	0.44
	*OPA1*	1.0 ± 0.11	1.0 ± 0.03	1.0 ± 0.04	1.0 ± 0.05	0.79

Protein levels of fission-associated protein *DNM1L* were assessed in human peripheral lung tissue from non-COPD patients (n = 6) and COPD patients (n = 10; GOLDII n = 3/10, GOLDIV n = 7/10) by western blot analysis. In addition, mRNA expression of fission- and fusion-associated markers was analysed in human peripheral lung tissue from non-COPD patients (n = 5) and COPD patients (n = 9; GOLDII n = 4/9, GOLDIV n = 5/9). Data are presented as mean fold change of COPD patients (GOLDII or GOLDIV) relative to non-COPD patients ± SEM. Statistical differences between COPD patients *versus* non-COPD patients (two groups) were tested using a two-tailed unpaired parametric t-test (normal distribution), or an unpaired nonparametric Mann–Whitney test (non-normal distribution) (p-value shown in last column). In addition, in case of testing the difference between COPD GOLDII or GOLDIV patients *versus* non-COPD patients (three groups), we used a one-way ANOVA and a Dunnett’s post-hoc test for multiple comparisons (normal distribution) or a Kruskal–Wallis test followed by a Dunn’s multiple comparisons test (non-normal distribution) (p-value not shown).

## Discussion

**Figure 5:**
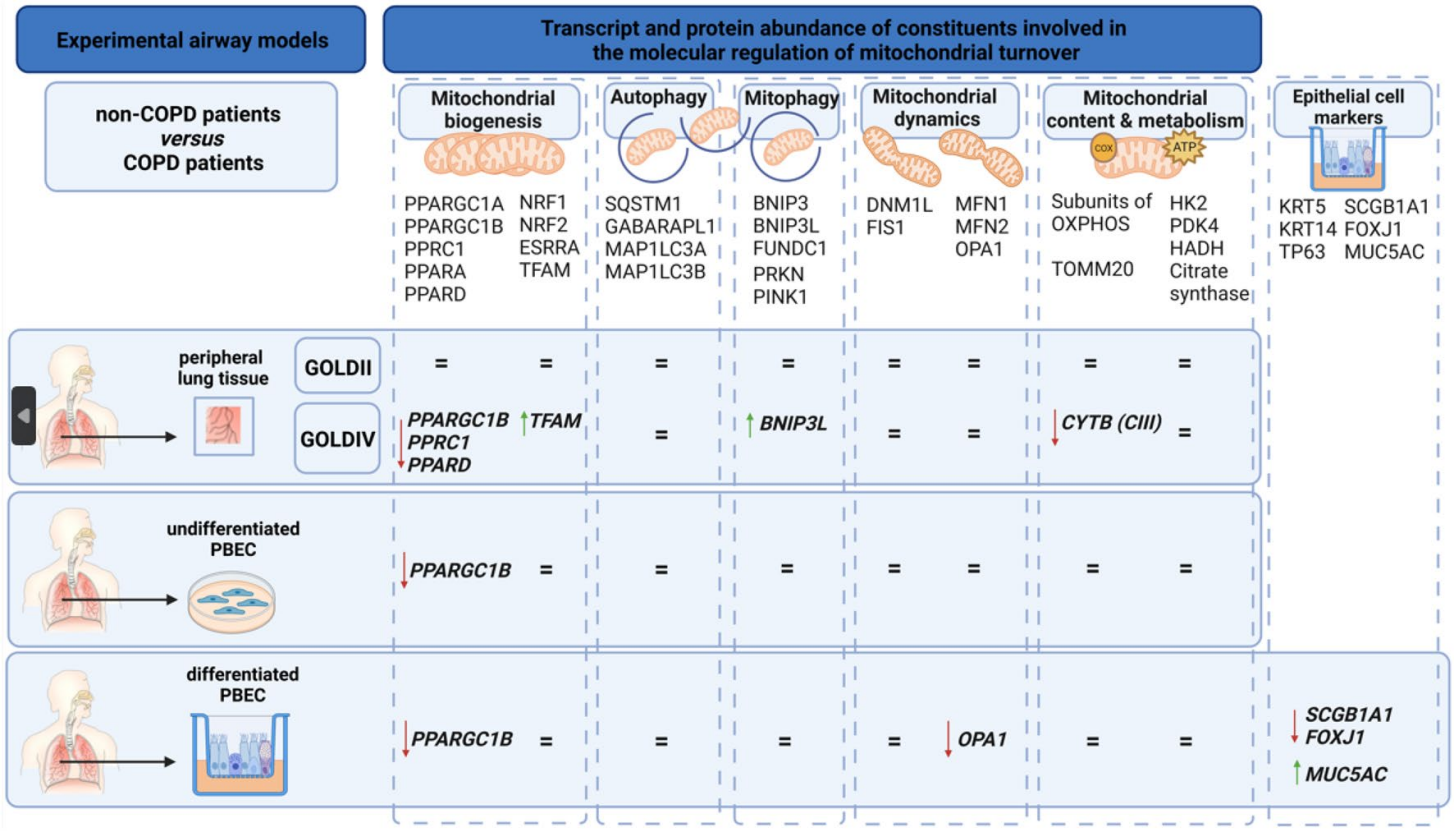
Overview of all data in the different models.

Abnormalities in mitochondrial morphology and content have been described in PBECs from COPD patients. However, whether or not the molecular pathways controlling this (mitochondrial biogenesis *versus* mitophagy) are altered, thus far was unknown. Moreover, if these changes also occur in peripheral lung tissue is unexplored. Therefore, in this study, as our primary aim, we evaluated for the first time, the protein and transcript abundance of a broad spectrum of molecules involved in the regulation of mitochondrial biogenesis, mitophagy, mitochondrial dynamics and mitochondrial metabolism in both peripheral lung tissue from COPD patients at different stages of the disease and in undifferentiated and differentiated PBEC from COPD patients and non-COPD patients. As our main findings, we observed a significant reduction in transcript levels of molecules controlling mitochondrial biogenesis and an upregulation of a molecule involved in receptor-mediated mitophagy (*BNIP3L)*, in peripheral lung tissue from very severe COPD patients. In general, these changes were not recapitulated in PBEC from COPD patients, with the exception of decreased *PPARGC1B* expression which was observed in both PBEC models. An overview of all data is presented in Fig. 5.

Our key observation was a significant decrease in transcript levels of molecules essentially involved in mitochondrial biogenesis in peripheral lung tissue and PBECs from COPD patients, especially in a very severe disease stage. Although the investigation of a comprehensive set of regulatory molecules involved in mitochondrial biogenesis has not been previously described, specific subsets of regulatory molecules have been investigated in PBEC or lung homogenates from COPD patients previously. In line with our data, progressively decreased expression of PPARGC1A was shown in lung homogenates of moderate and severe COPD patients (GOLDII and GOLDIII), while increased expression levels were observed in mild COPD patients (GOLDI) compared to control subjects^38^. In contrast, increased transcript levels of *PPARGC1A* have been observed in PBEC of ex-smoking COPD GOLDIV patients relative to never-smoking or smoking controls^19^. Regarding the impact of cigarette smoke on the regulation of mitochondrial biogenesis, higher mRNA levels of *PPARGC1A* and *PPRC1* and lower mRNA levels of *PPARGC1B* have been reported differentiated PBECs upon acute whole CS-exposure^39^. Similar findings were reported in in bronchial epithelial cells s exposed to CS extract^40^. In addition, decreased expression of PPARGC1A has been observed in experimental models of CS-induced COPD, e.g., mice instilled with CS extract^41^ or in mice instilled with lipopolysaccharide and CS^42^, while elevated abundance of PPARGC1A have been observed in rats treated with CS+ lipopolysaccharide^38^.

In addition, in our study, a key molecule involved in transcription of the mitochondrial genome, *TFAM*, was found to be increased in peripheral lung tissue from very severe COPD patients compared to non-COPD patients. These differences were previously not observed in lung homogenates from ex-smoking severe COPD GOLDIV patients relative to never-smoking controls^19^. Moreover, in contrast to our findings, decreased TFAM levels were previously observed in lung tissue from COPD patients compared to non-COPD patients^43^. These apparently contradictory findings i could be partly explained by differences in disease status of these study populations. Also, as we previously reported that CS can increase *Tfam* mRNA expression^39^, an impact of smoke exposure on the abundance of these transcription factors cannot be excluded. In this context, increased expression levels may be evidence of a compensatory cellular response to induce mitochondrial biogenesis.

Although the directionality of these alterations may depend on the cell type, disease status or time or dose of exposure, this collective body of evidence demonstrates that the molecular regulation of mitochondrial biogenesis is disturbed in COPD patients and is impacted by smoking in epithelial cells of both the bronchial and alveolar compartment. The fact that some of our read-out parameters correlated (inversely) with lung function parameters suggests that the regulation of mitochondrial metabolism/turnover may be important for maintaining adequate lung function or that impairments in (the regulation of) mitochondrial content/function may be implicated in deterioration of lung function in COPD. These notions have been suggested in literature previously^18,44,45^.

For ubiquitin-mediated mitophagy, we found no significant alterations in the abundance of any of the molecules involved in these pathways. In contrast, it has been previously described that PINK1 expression was elevated in lung tissue homogenates from COPD patients^19,46,47^. In addition, a downregulation of PRKN protein was observed in lung homogenates from COPD patients compared to non-COPD smokers, along with a positive correlation between PRKN protein levels and percentage FEV_1_/FVC^46^. These alterations would suggest aberrant ubiquitin-mediated mitophagy in COPD. Moreover, decreased PRKN protein levels were shown smokers relative to non-smokers as well as in mitochondria isolated from mice lung exposed to CS for 6 months indicating impaired PRKN translocation to mitochondria^48^. Increased *PINK1* and decreased *PRKN* expression was also observed in PBEC cultures acutely exposed to CS^39^. These data imply a role for smoke exposure in changes in ubiquitin-mediated mitophagy in COPD.. The reason for a lack of alterations in the abundance of these molecules in our study is unclear but disease severity or smoking history may play a role. In addition, the relevance and exact implication of potential changes in this mitophagy pathway in COPD pathogenesis remains to be established, however it is likely that ubiquitin-mediated mitophagy may play a role as it has been shown that animals deficient for PRKN display an increased susceptibility for developing CS-induced emphysema^49^.

Next to ubiquitin-mediated mitophagy, we also explored other known mitophagy pathways. In this regard, we observed increased mRNA levels of receptor-mediated mitophagy-associated protein *BNIP3L* in peripheral lung tissue from very severe COPD patients. To our knowledge, this is the first time this has been investigated in airway tissues/cells from COPD patients. Interestingly, we recently showed that the abundance of proteins and genes associated with receptor-mediated mitophagy were increased in PBECs following acute exposure to CS^39^ which is in line with several other in vitro and in vivo studies^50,51^. CS-induced hypoxia may play a role in mediating this as previous studies showed that CS stimulates hypoxia-related signalling in airway cell models^52,53^ which is a known activating pathway for receptor-mediated mitophagy/autophagy^54–59^.

Interestingly, we observed altered gene expression profiles suggestive of impaired ciliogenesis and increased goblet cell formation in COPD PBEC cultures compared to non-COPD cultures. Alterations in the composition of these cell populations have been previously observed in the airway epithelium of COPD patients and smokers and in vitro in small airway epithelial cells exposed to CS. Changes observed herein included an increased number of basal and secretory cells, impaired length/number of ciliated cells, and metaplasia of squamous cells^7–10,60^. Both smoking and the disease itself has been suggested to be a contributing factor to these changes in COPD patients^7^.

No differences were observed in TEER development after differentiation of PBECs between COPD and non-COPD patients. It should be noted however that TEER is just an indication of barrier integrity and not for cellular composition or functionality of the epithelial layer. Some studies showed dysfunctional cilia in PBECs from COPD patients^11,12^ and reduced beating frequency was shown in nasal cilia from COPD patients^13^. Also, a significant association has been found between mucus hypersecretion and lung function decline in COPD patients^14^. The exact pathways underlying this are unknown, but there may be a role for mitochondrial dysfunction herein. We performed correlation analyses to explore correlations between the abundance of mitochondrial markers and markers associated with muco-ciliary function in differentiated PBEC from COPD *versus* non-COPD patients, but failed to observe convincing correlations. Further research into the relationship between mucociliary function, mucus secretion, and the role of mitochondrial dysfunction herein is required in COPD patients.

Obviously, our study has limitations. Firstly, obtaining and analyzing both undifferentiated- and differentiated PBEC cultures as well as peripheral lung tissue from the same (non)COPD patients would have been ideal. Unfortunately, we were limited to retrieving our biological material from 3 different patient cohorts, which likely introduced variability in our results. Secondly, it must be noted that the peripheral lung tissue as well as non-COPD PBECs used for the undifferentiated PBEC model were isolated from subjects undergoing lung cancer resection surgery. Although material was collected from locations as distant as possible from the tumor, obviously these subjects were not healthy which may have influenced our results. Thirdly, changes in the regulatory pathways that we describe are not necessarily reflective of actual mitochondrial function. Lastly, our 2 of our 3 models or biological material consisted of multiple different cell types, obscuring cell-type specific responses^61,62^. Future studies using microdissection of the lung tissue or singe-cell sequencing, as well as (co-)cultures of several cell populations may contribute to the understanding of the regulation of mitochondrial turnover and metabolism and the involvement of specific cell types in the context of COPD.

Another observation from our study is the discrepancy in transcript *versus* protein expression in peripheral lung tissue from non-COPD patients and COPD patients. Whether these discrepancies can be explained by (smoking-induced) changes in post-transcriptional^63^, remains to be established. Additional explanations could be the low number of patients included in the study and the biological variation. With regard to discrepancies in mRNA and protein levels of mitophagy constituents, the fact that mitophagy proteins are actively broken down together with the mitochondrion during mitophagy and need to be replenished (by transcriptional) upregulation of the respective genes, may contribute to apparently discrepant findings. This also warrants caution in the interpretation of these findings with regard to activation status of the mitophagy process.

In addition to these considerations, some differences in gender and age were identified between moderate and very severe COPD patients that we included in our study. The observation of a more pronounced downregulation in the expression of regulators controlling mitochondrial biogenesis in very severe COPD patients (female; younger age) *versus* moderate COPD patients (male; older age), is in line with a recent study reporting that genes related to mitochondrial biogenesis/function were differently expressed in younger and older smokers, and in COPD patients compared to non-smokers^64^. Interestingly, recent evidence also supports a role for accelerated ageing and related mitochondrial dysfunction in the pathogenesis of COPD^17^. Furthermore, other studies also point to a significant impact of gender on mitochondrial pathways^22,65,66^.

As a final point, it must be emphasized that our observations are descriptive in nature and do not imply causal involvement of changes in these pathways in COPD pathogenesis. As a decrease in PPARGC1B was the most consistent finding in our study, we attempted to address any causal implication by knocking down of this protein in non-COPD PBECs but unfortunately failed to achieve sufficient knock-down. Difficulties knock-down in PBECs have been described previously^67^. However, based on literature it can be speculated that, given the well-known role of PPARGC1B (and its better known family member PPARGC1A) in control of mitochondrial function/content, loss of this co-activator molecule likely leads to loss of mitochondrial functionality and content^26–28^. In addition, although mitochondrial abnormalities have been described in PBECs and now also in peripheral lung tissue of COPD patients, whether or not these are involved in COPD pathogenesis is unclear. In this context however, it is interesting to note that mitochondrial dysfunction is well-known to lead to oxidative stress, inflammation, cell death and senescence, all of which are hallmark features in cells of the airways and lungs in COPD. Also, the role of mitochondrial dysfunction in the pathogenesis of lung disease, including COPD, has been extensively reviewed previously^18,19^. Moreover, using a mouse model of COPD (smoking mice) Cloonan et al. very elegantly showed that preventing cigarette smoke-induced mitochondrial dysfunction ameliorated COPD features in these animals which does suggest causal involvement of mitochondrial dysfunction in COPD pathogenesis^44^.

In conclusion, our study shows changes in the expression of genes controlling mitochondrial biogenesis and receptor-mediated mitophagy in peripheral lung tissue from very severe COPD patients, along with a correlation with lung function. In general, these results were not observed in undifferentiated and differentiated PBEC from COPD patients, except decreased *PPARGC1B* transcript levels in both PBEC models. If these changes are involved in the airway pathophysiology of COPD, contribute to aberrant mitochondrial function or may represent compensatory mechanisms remains to be established.

### Supplementary Information

Supplementary Information 1.

Supplementary Information 2.

## Data Availability

The datasets used and/or analysed during the current study are available from the corresponding author on reasonable request.

## Abbreviations

ACTB: Actin B
AEGM: Airway epithelial cell growth medium
ALI: Air–liquid interface
ATP5F1A: ATP synthase F1 subunit alpha
B2M: Beta-2 microglobulin,
BEGM: Bronchial epithelial cell growth medium
BSA: Bovine serum albumin
BMI: Body mass index
BNIP3: BCL2 interacting protein 3
BNIP3L: BCL2 Interacting protein 3-Like
COPD: Chronic obstructive pulmonary disease
CS: Cigarette smoke
COXII: Cytochrome C oxidase subunit II
COXIII: Cytochrome C oxidase subunit III
COXIV: Cytochrome C oxidase subunit 4I1
CYC1: Cytochrome C-1
CYTB: Mitochondrially encoded cytochrome B
DNM1L: Dynamin-1-Like
ESRRA: Estrogen related receptor, alpha
FEV1: Forced expiratory volume in first second
FIS1: Fission, mitochondrial 1
FOXJ1: Forkhead box J1
FUNDC1: FUN14 domain containing 1
FVC: Forced vital capacity
GABARAPL1: GABA type a receptor associated protein like 1
HADHA: Hydroxyacyl-CoA dehydrogenase trifunctional multienzyme complex subunit alpha
HBSS: Hank’s balanced salt solution
HK2: Hexokinase 2
KRT5: Keratin 5
KRT14: Keratin 14
MAP1LC3A: Microtubule-associated protein 1 light chain 3 alpha
MAP1LC3B: Microtubule-associated protein 1 light chain 3 beta
MFN1: Mitofusin 1
MFN2: Mitofusin 2
MUC5AC: Mucin 5AC, oligomeric mucus/gel-forming
NDUFB3: NADH:Ubiquinone oxidoreductase subunit B3
NDUFB8: NADH:Ubiquinone oxidoreductase subunit B8
NRF1: Nuclear respiratory factor 1,
NRF2: Nuclear respiratory factor 2
OPA1: OPA1, Mitochondrial dynamin like GTPase
OXPHOS: Oxidative phosphorylation
PBEC: Primary bronchial epithelial cells
PDK4: Pyruvate dehydrogenase kinase 4
PINK1: PTEN Induced kinase 1
PPAR: Peroxisome proliferator-activated receptor
PPARA: Peroxisome proliferator activated receptor alpha
PPARD: Peroxisome proliferator activated receptor delta
PPARG: Peroxisome proliferator-activated receptor gamma
PPARGC1: Peroxisome proliferator-activated receptor gamma, coactivator 1
PPARGC1A: Peroxisome proliferator-activated receptor gamma, coactivator 1 Alpha
PPARGC1B: Peroxisome proliferator-activated receptor gamma, coactivator 1 beta
PPIA: Peptidylprolyl isomerase A
PPRC1: Peroxisome proliferator-activated receptor gamma, coactivator-related 1
PRKN: Parkin RBR E3 ubiquitin protein ligase
RPL13A: Ribosomal protein L13A
SCGB1A1: Secretoglobin family 1a member 1
SDHB: Succinate dehydrogenase complex iron sulfur subunit B
SEM: Standard error of the mean
SQSTM1: Sequestosome 1, transcript variant 1/4
TBST: Tween20 Tris-buffered saline
TEER: Transepithelial electrical resistance
TFAM: Transcription Factor A, mitochondrial
TOMM20: Translocase of outer mitochondrial membrane 20
TP63: Tumor protein P63
UQCRC2: Ubiquinol-cytochrome C reductase core protein 2
