# Supplementary material for: Alterations in the molecular control of mitochondrial turnover in COPD lung and airway epithelial cells
Source: Sci Rep. 2024 Feb 27;14:4821. doi: 10.1038/s41598-024-55335-8 (PMC10899608; doi:10.1038/s41598-024-55335-8)

Western Blot overview

Alterations in the transcript abundance of molecules controlling mitochondrial turnover in peripheral lung tissue from chronic obstructive pulmonary disease patients

1) Peripheral lung tissue from non-COPD and COPD patients

Figure S1.

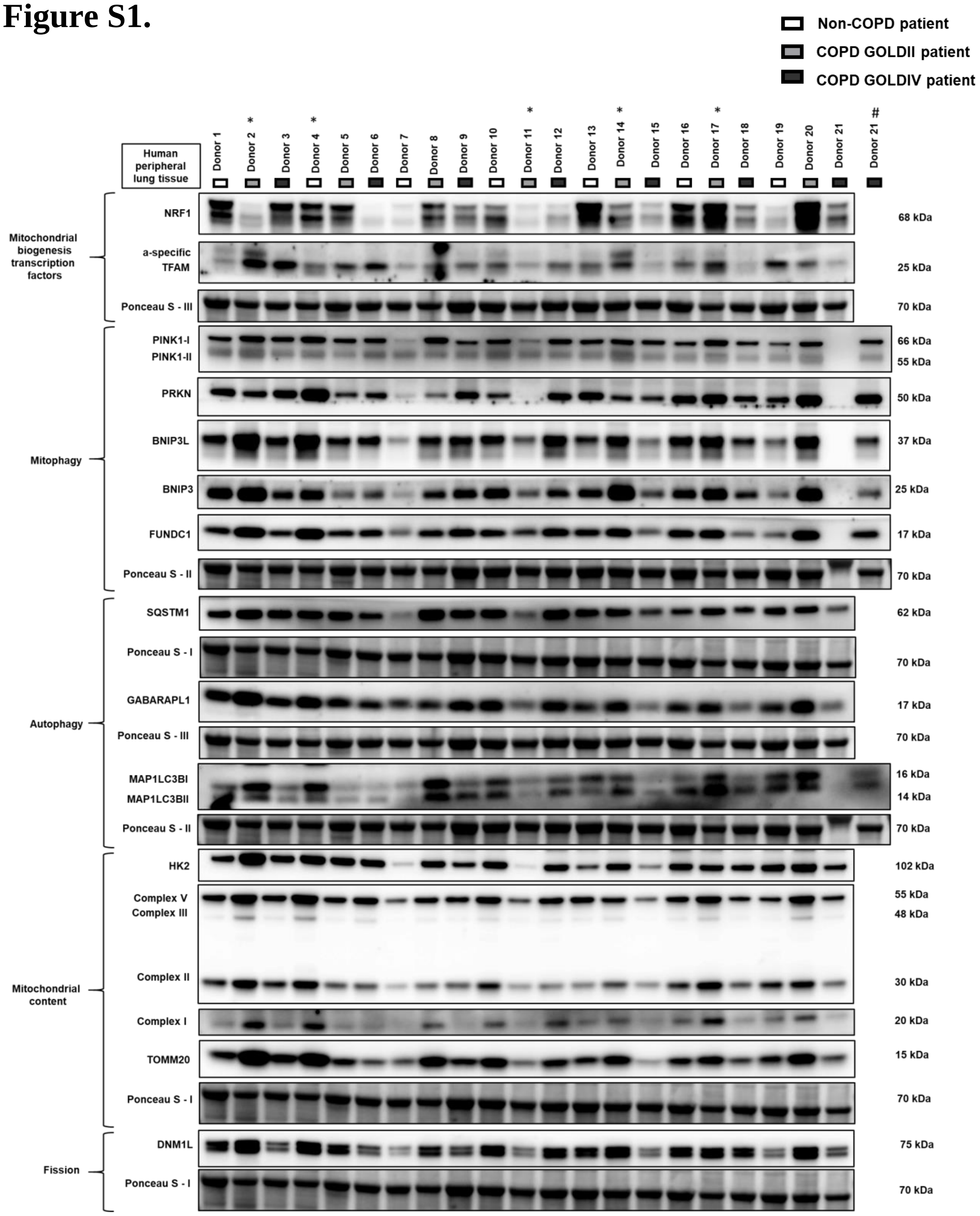

Ponceau S-I

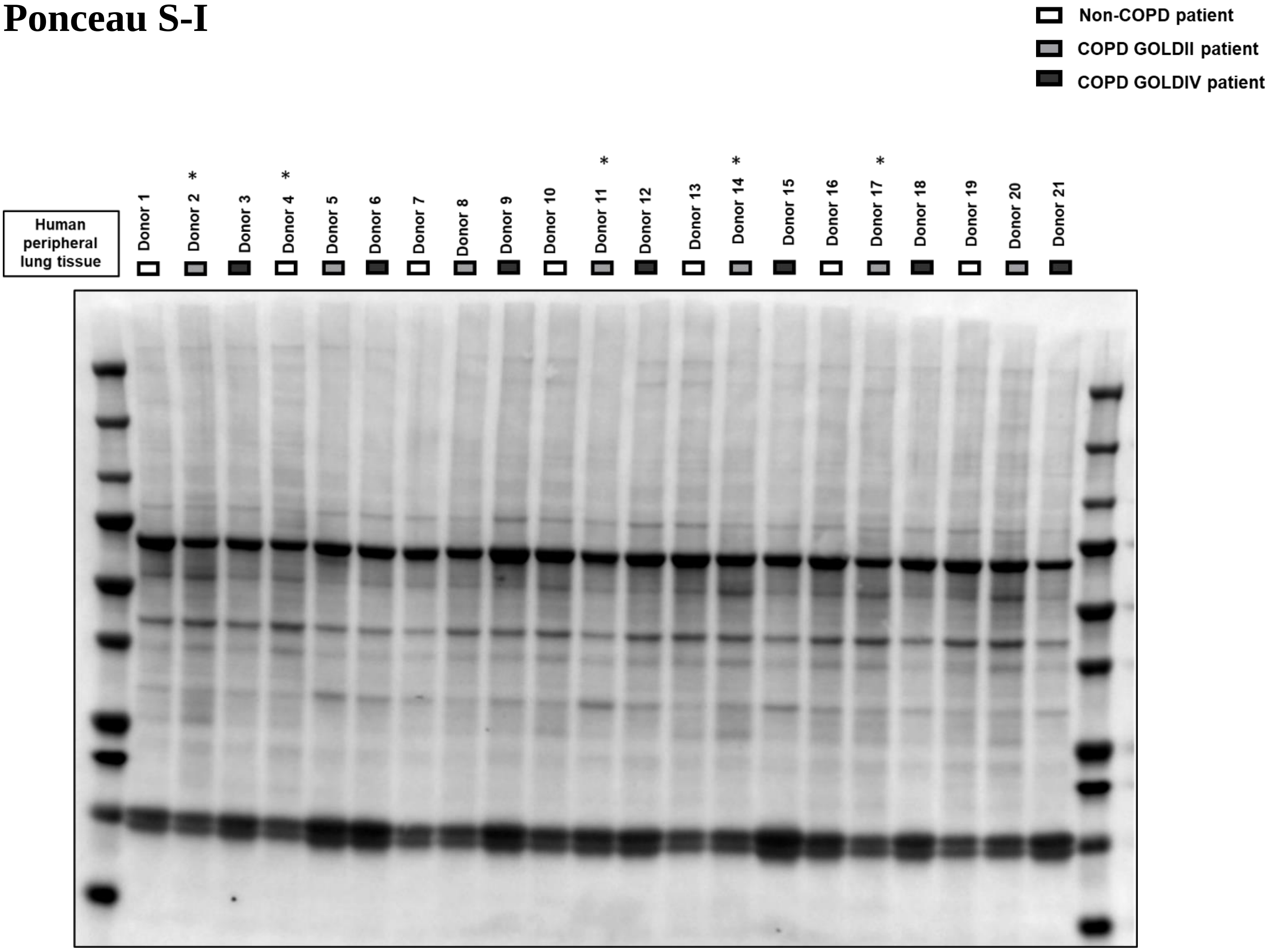

Non-COPD patient
COPD GOLDII patient
COPD GOLDIV patient
Human peripheral lung tissue
Donor 1
Donor 2 *
Donor 3
Donor 4 *
Donor 5
Donor 6
Donor 7
Donor 8
Donor 9
Donor 10
Donor 11 *
Donor 12
Donor 13
Donor 14 *
Donor 15
Donor 16
Donor 17 *
Donor 18
Donor 19
Donor 20
Donor 21

Ponceau S-II

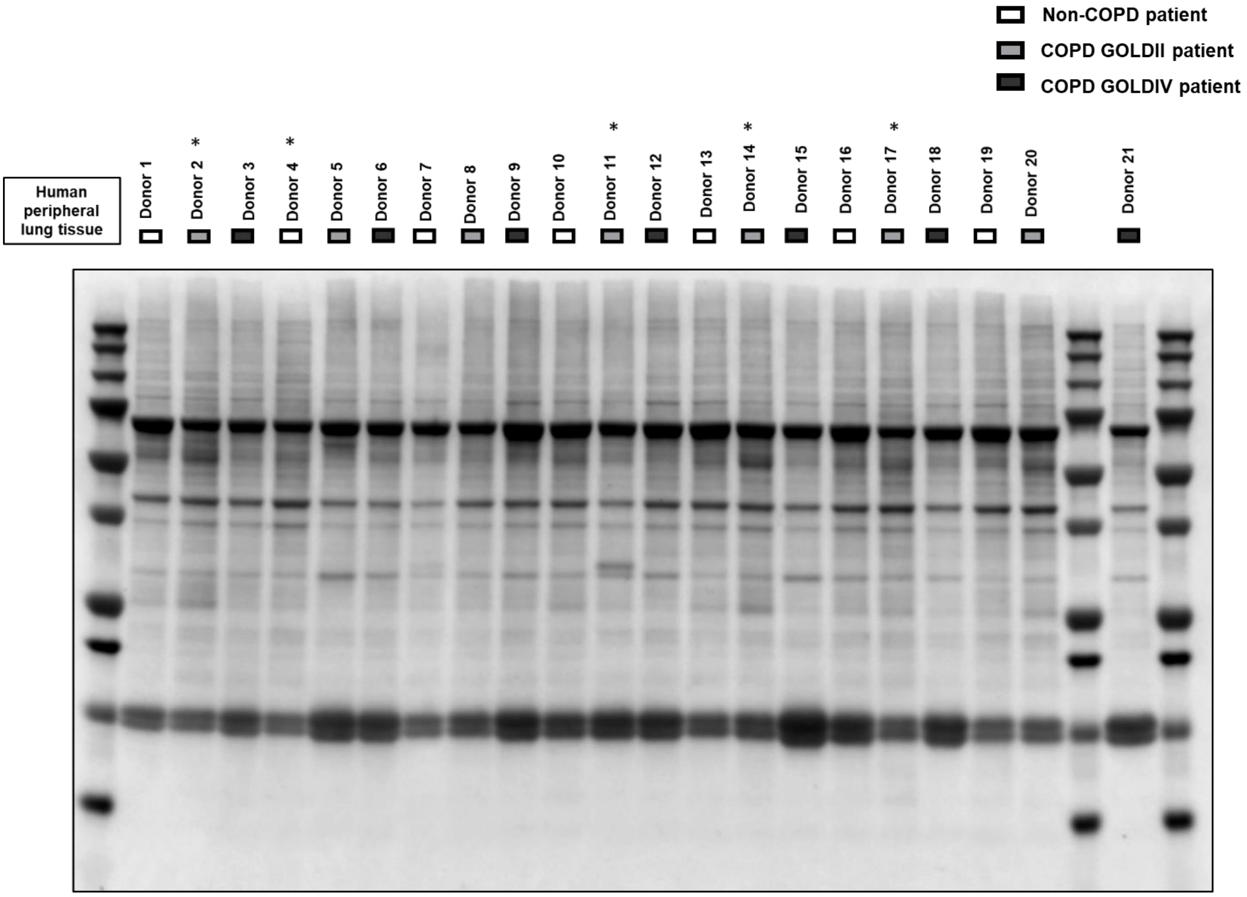

Non-COPD patient
COPD GOLDII patient
COPD GOLDIV patient
Human peripheral lung tissue
Donor 1
Donor 2 *
Donor 3
Donor 4 *
Donor 5
Donor 6
Donor 7
Donor 8
Donor 9
Donor 10
Donor 11 *
Donor 12
Donor 13
Donor 14 *
Donor 15
Donor 16
Donor 17 *
Donor 18
Donor 19
Donor 20
Donor 21

Ponceau S-III

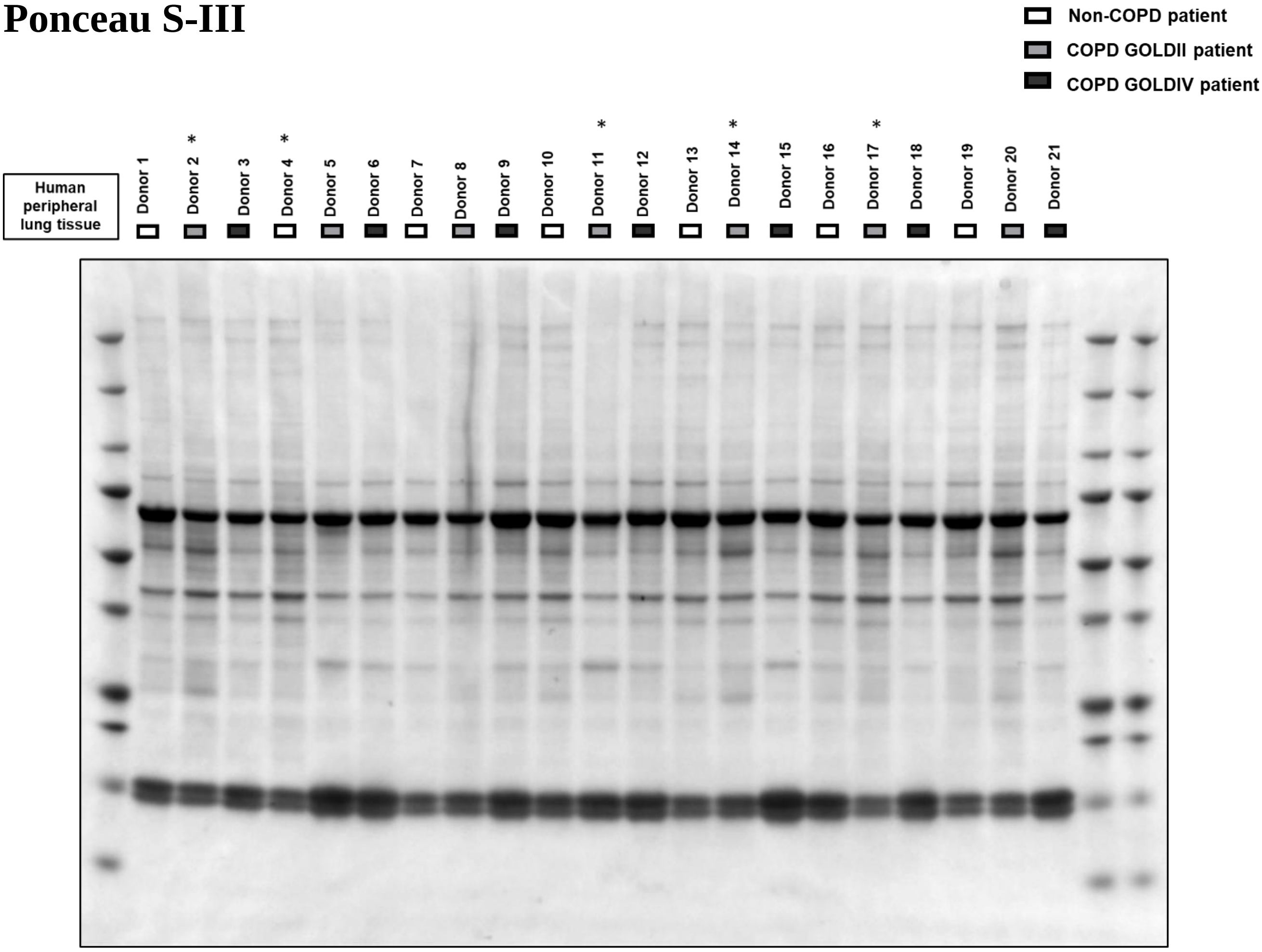

2) Undifferentiated PBEC from non-COPD and COPD patients

Figure S2.

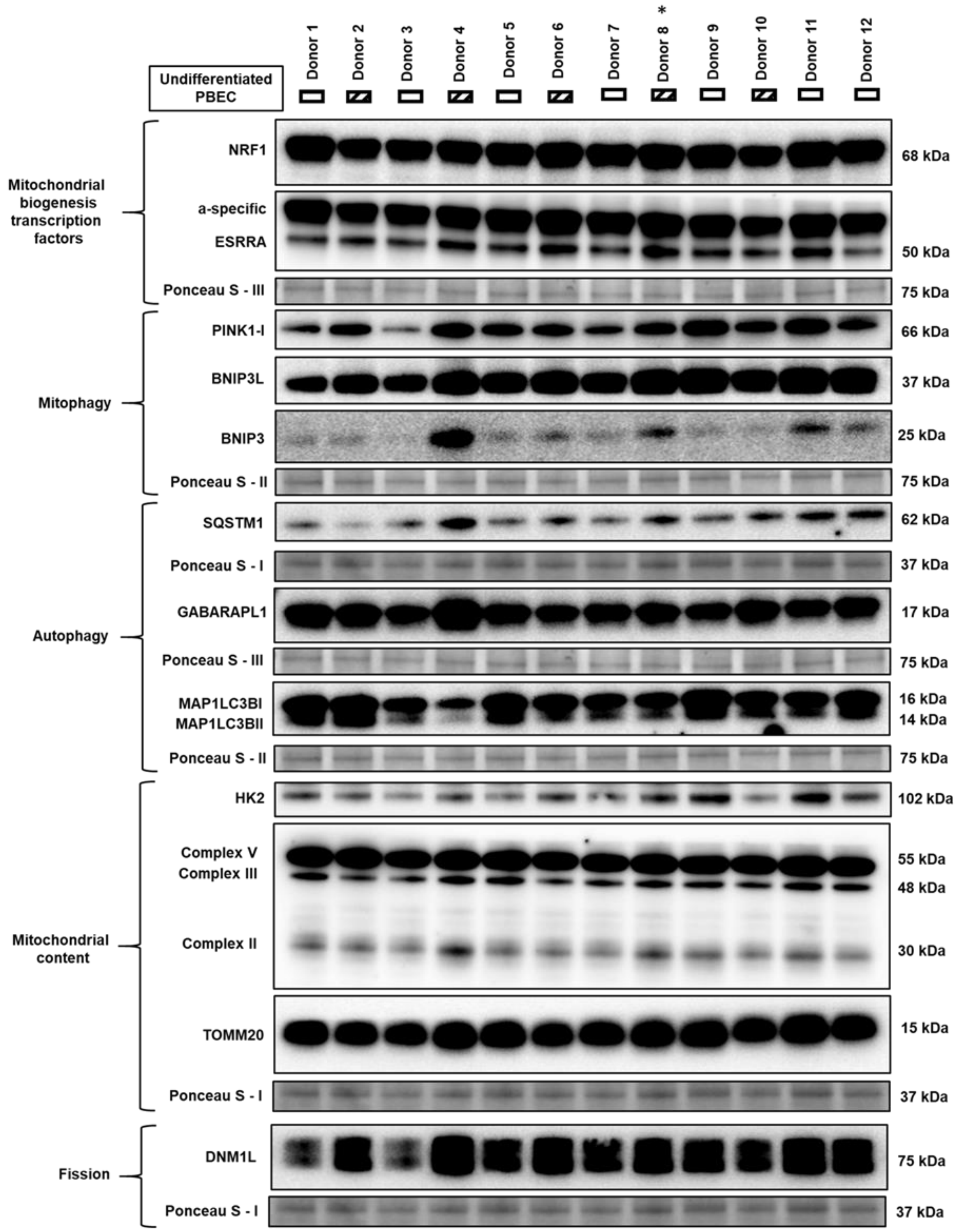

Ponceau S-I

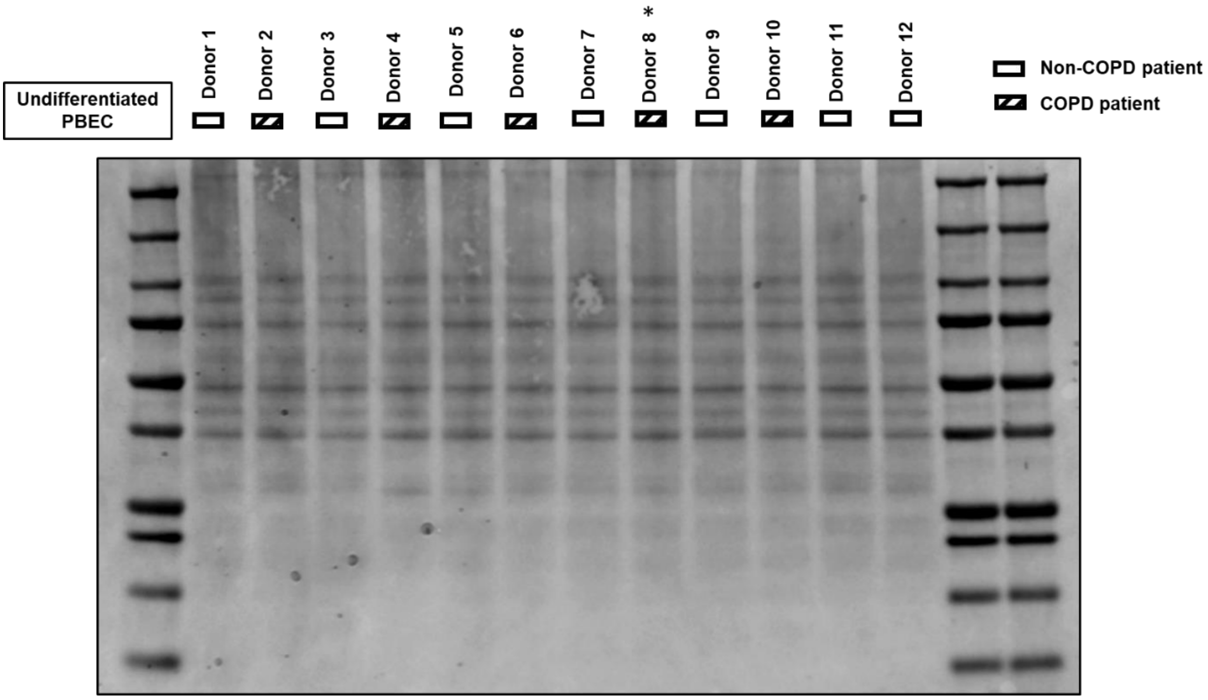
Undifferentiated PBEC
Donor 1
Donor 2
Donor 3
Donor 4
Donor 5
Donor 6
Donor 7
Donor 8 *
Donor 9
Donor 10
Donor 11
Donor 12
Non-COPD patient
COPD patient

Ponceau S-II

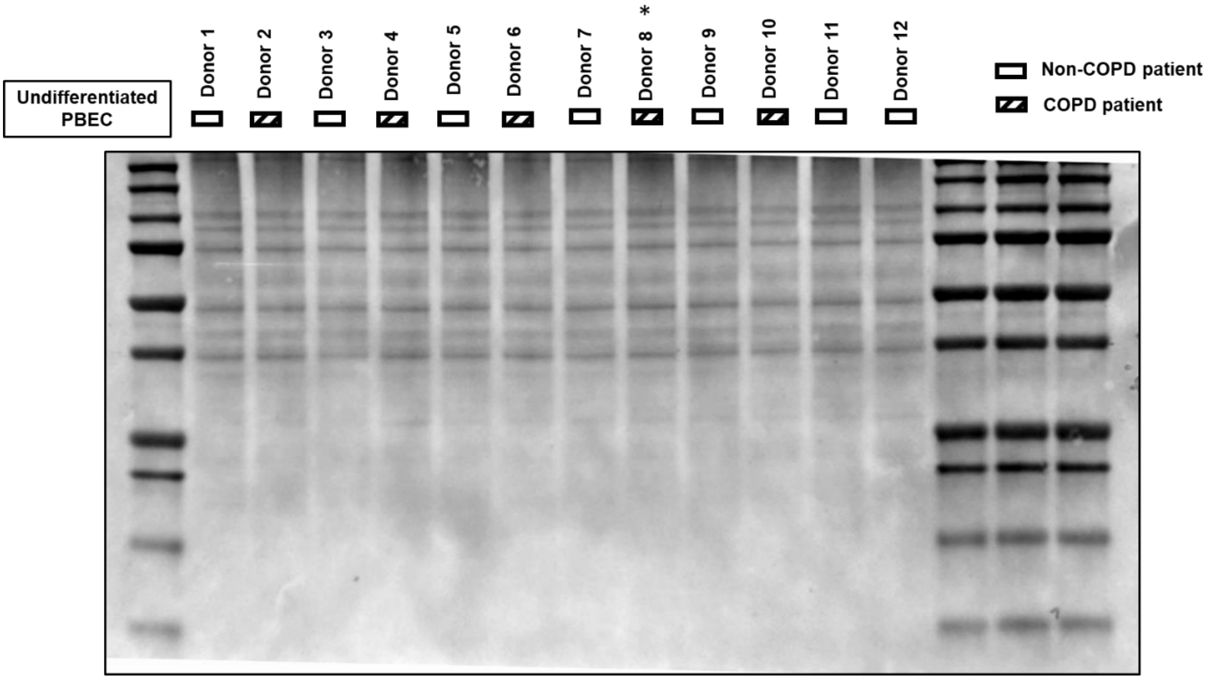
Undifferentiated PBEC
Donor 1
Donor 2
Donor 3
Donor 4
Donor 5
Donor 6
Donor 7
Donor 8 *
Donor 9
Donor 10
Donor 11
Donor 12
Non-COPD patient
COPD patient

Ponceau S-III

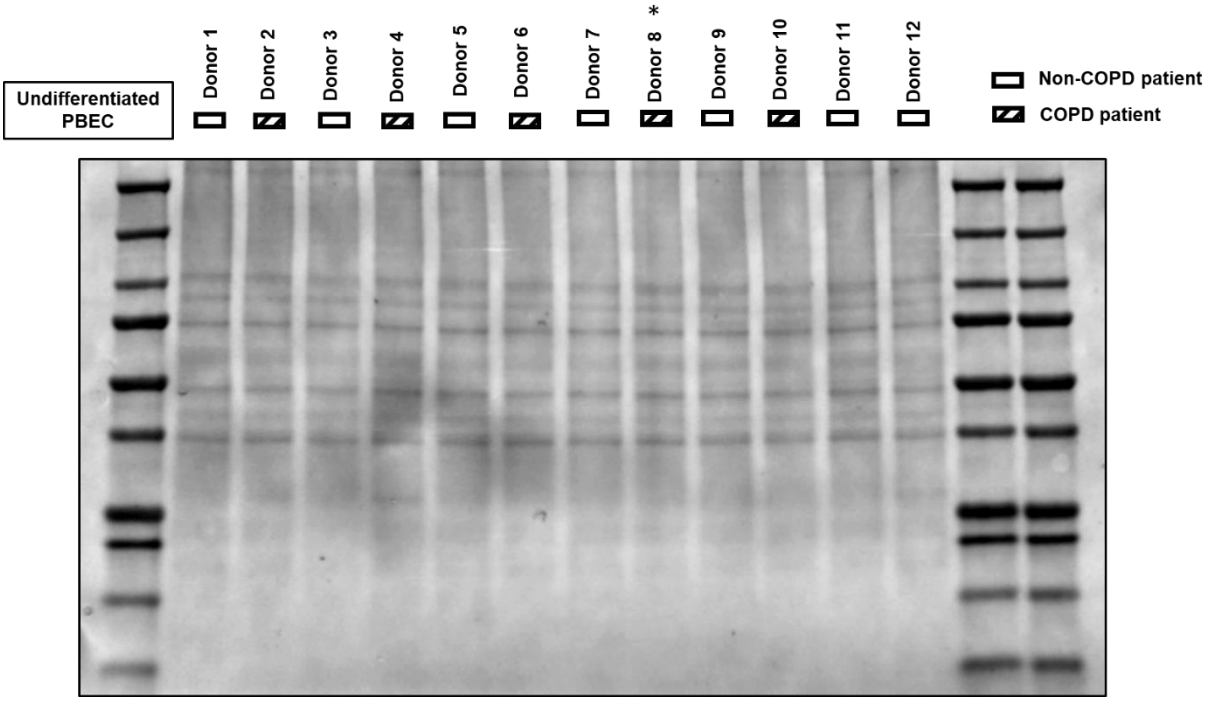

3) Differentiated PBEC from non-COPD and COPD patients

Figure S3.

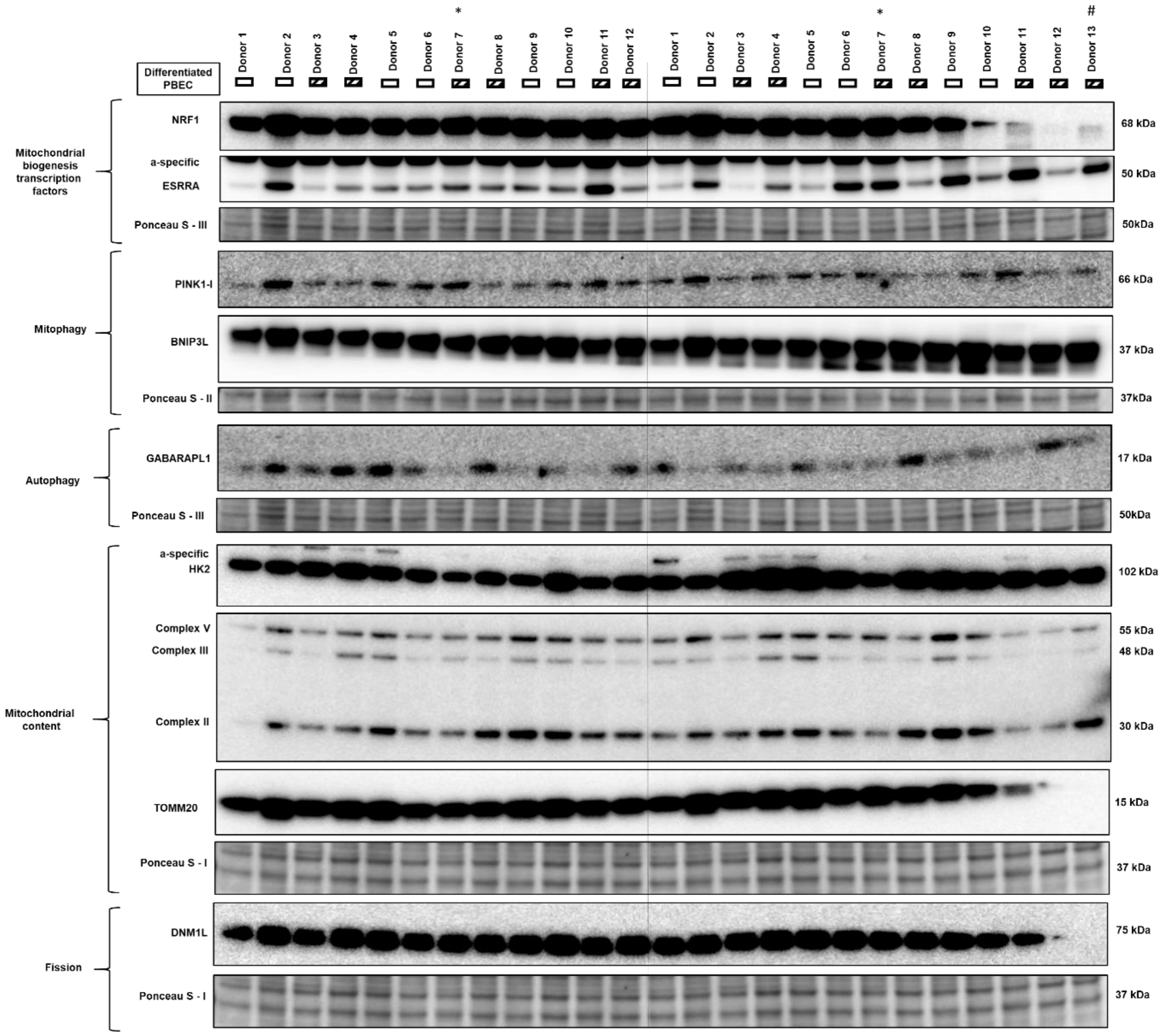

Ponceau S-I

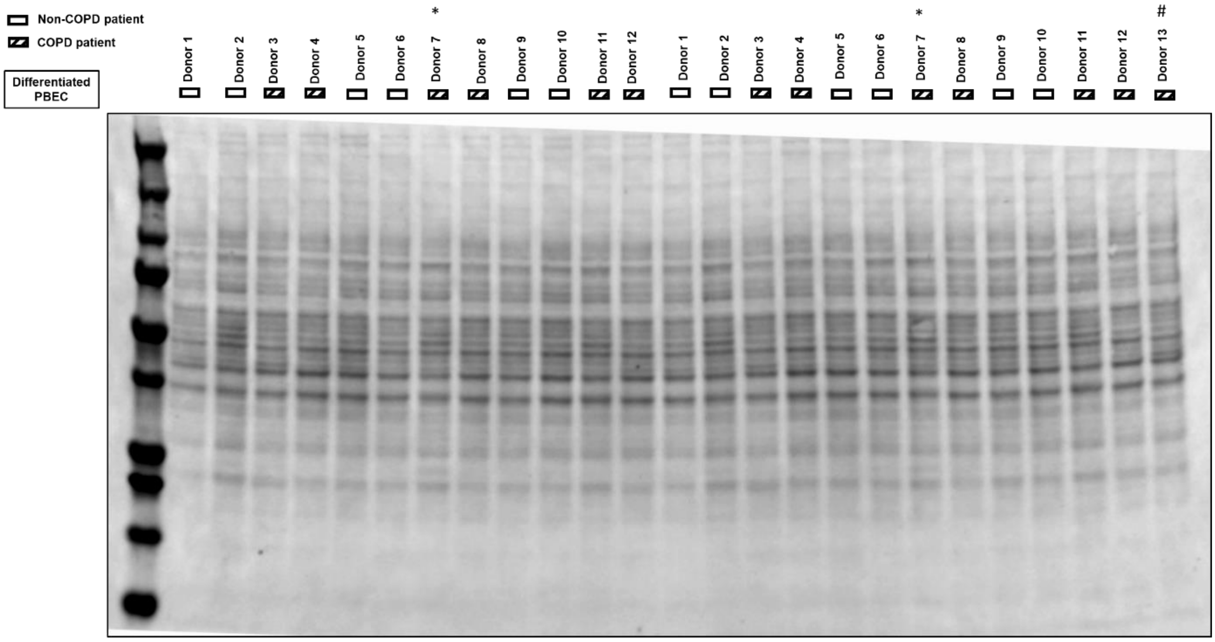
Non-COPD patient
COPD patient
Differentiated PBEC
Donor 1
Donor 2
Donor 3
Donor 4
Donor 5
Donor 6
Donor 7
Donor 8
Donor 9
Donor 10
Donor 11
Donor 12
Donor 1
Donor 2
Donor 3
Donor 4
Donor 5
Donor 6
Donor 7
Donor 8
Donor 9
Donor 10
Donor 11
Donor 12
Donor 13
*
*
#

Ponceau S-II

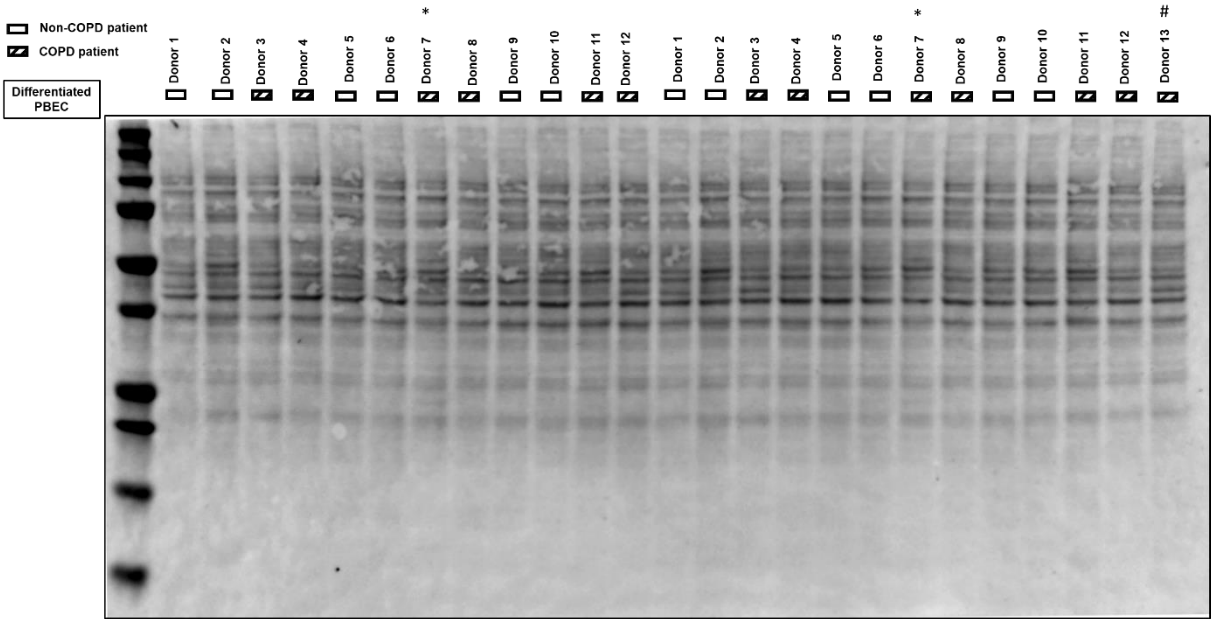
Non-COPD patient
COPD patient
Differentiated PBEC
Donor 1
Donor 2
Donor 3
Donor 4
Donor 5
Donor 6
*
Donor 7
Donor 8
Donor 9
Donor 10
Donor 11
Donor 12
Donor 1
Donor 2
Donor 3
Donor 4
Donor 5
Donor 6
*
Donor 7
Donor 8
Donor 9
Donor 10
Donor 11
Donor 12
#
Donor 13

Ponceau S-III

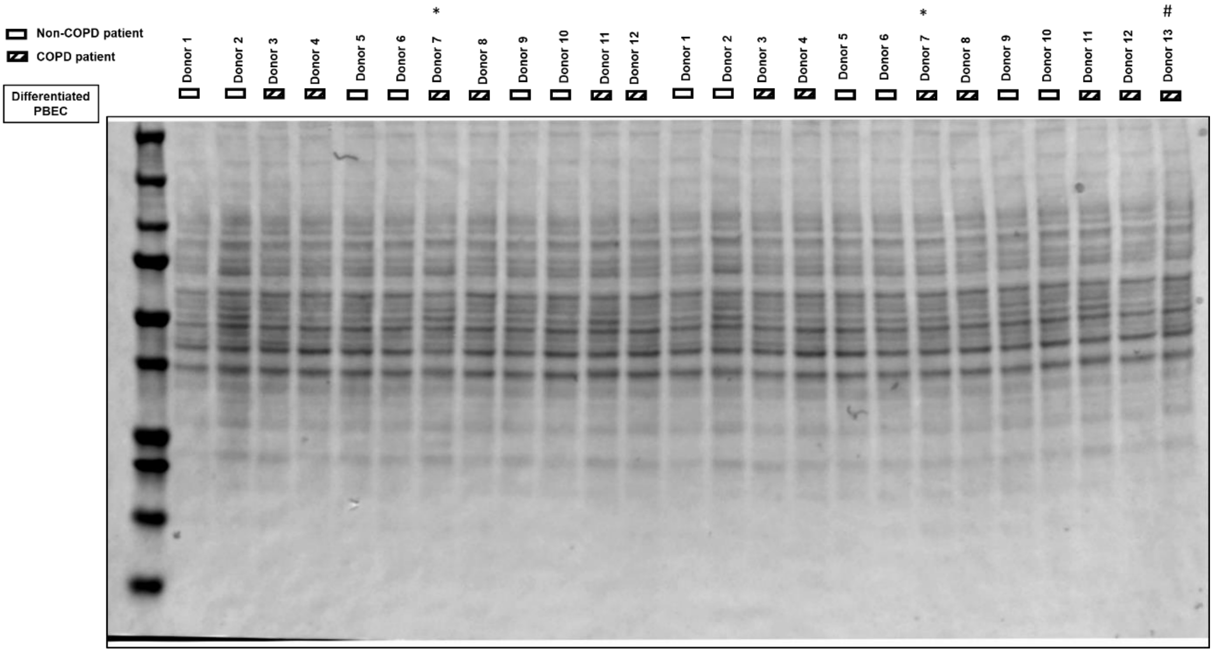

Supplement: Supplementary file 1 — Supplementary Information 1. [file 41598_2024_55335_MOESM1_ESM.pdf]
