# Supplementary material for: Alterations in the molecular control of mitochondrial turnover in COPD lung and airway epithelial cells
Source: Sci Rep. 2024 Feb 27;14:4821. doi: 10.1038/s41598-024-55335-8 (PMC10899608; doi:10.1038/s41598-024-55335-8)
Supplement: Supplementary file 2 — Supplementary Information 2. [file 41598_2024_55335_MOESM2_ESM.docx]

**Supplementary data**

**Alterations in the molecular control of mitochondrial turnover in COPD lung and airway epithelial cells**

Tulen, Christy B.M.^1^, van de Wetering, Cheryl^2^, Schiffers, Caspar H.J.^2^, Weltjens, Ellen ^1^ Benedikter, Birke J.^3,4^, Leermakers, Pieter A.^1^, Boukhaled, Juliana H.^1^, Drittij, Marie-José^1^, Schmeck, Bernd T.^4,5,6^, Reynaert, Niki L.^2,7^, Opperhuizen, Antoon^1,8^, van Schooten, Frederik-Jan^1^, Remels, Alexander H.V.^1^

^1^School of Nutrition and Translational Research in Metabolism (NUTRIM), Department of Pharmacology and Toxicology, Maastricht University Medical Center+, Maastricht, the Netherlands

^2^School of Nutrition and Translational Research in Metabolism (NUTRIM), Department of Respiratory Medicine, Maastricht University Medical Center+, Maastricht, the Netherlands

^3^School of Nutrition and Translational Research in Metabolism (NUTRIM), Department of Microbiology, Maastricht University Medical Center+, Maastricht, the Netherlands

^4^Institute for Lung Research, Philipps-University Marburg, Marburg, Germany, Member of the German Center for Lung Research (DZL), Universities of Giessen and Marburg Lung Center

^5^Department for Respiratory and Critical Care Medicine, Clinic for Respiratory Infections, University Medical Center Marburg, Marburg, Germany

^6^German Centers for Lung Research (DZL) and for Infectious Disease Research (DZIF), SYNMIKRO Center for Synthetic Microbiology, Philipps-University Marburg, 35037 Marburg, Germany

^7^Primary Lung Culture (PLUC) facility, Maastricht University Medical Center+, Maastricht, the Netherlands

^8^Office of Risk Assessment and Research, Netherlands Food and Consumer Product Safety Authority (NVWA), Utrecht, the Netherlands

The authors have declared no conflict of interest.

This research is supported by the Netherlands Food and Consumer Product Safety Authority (NVWA).

**Corresponding author:**

A.H.V. Remels

Mail: [a.remels@maastrichtuniversity.nl](mailto:a.remels@maastrichtuniversity.nl)

School of Nutrition and Translational Research in Metabolism (NUTRIM)

Department of Pharmacology and Toxicology, Maastricht University Medical Center+

Universiteitssingel 50, 6629 ER Maastricht, the Netherlands

**
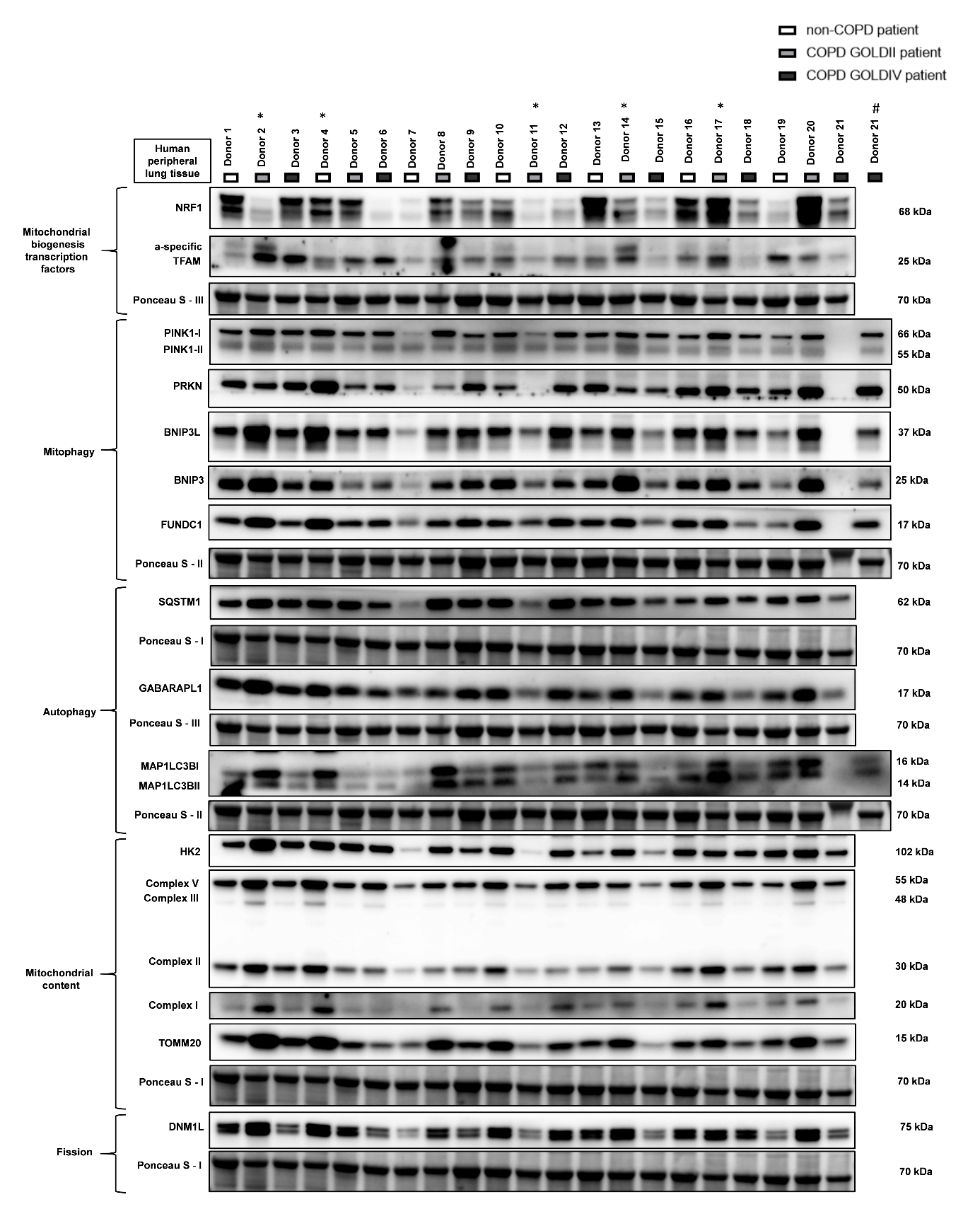
**

**Figure S1: Abundance of proteins involved in mitochondrial turnover and metabolism in peripheral lung tissue from COPD patients.** Protein abundance of transcription factors involved in mitochondrial biogenesis, as well as regulators associated with mitophagy, autophagy, mitochondrial content and fission in peripheral lung homogenates from non-COPD patients (n=6) and COPD patients (n=10; GOLDII n=4/10, GOLDIV n=6/10). A single sample per donor was analysed. All western blot images of the target proteins along with the corresponding normalization bands of the Ponceau S staining are shown of all patients/group. The Ponceau S band (70 kDa) displayed is representative for the total Ponceau S staining per sample. Because several proteins of interest were detected on a similar gel, which is possible due to their difference in molecular mass, correction for total protein content/loading of these target proteins was based on the same Ponceau S staining. To specify, Gel I: HK2, DNM1L, OXPHOS, SQSTM1, TOMM20; Gel II: BNIP3L, BNIP3, FUNDC1, PINK1, PRKN, MAP1LC3B; Gel III: NRF1, TFAM, GABARAPL1. ^*^These subjects were excluded post-analysis based on box plot analysis (i.e., values more than three interquartile ranges from the end of a box) in IBM Statistics SPSS 25. ^#^Due to technical issues, sample of donor 21 was loaded in a different lane on GELII.

**
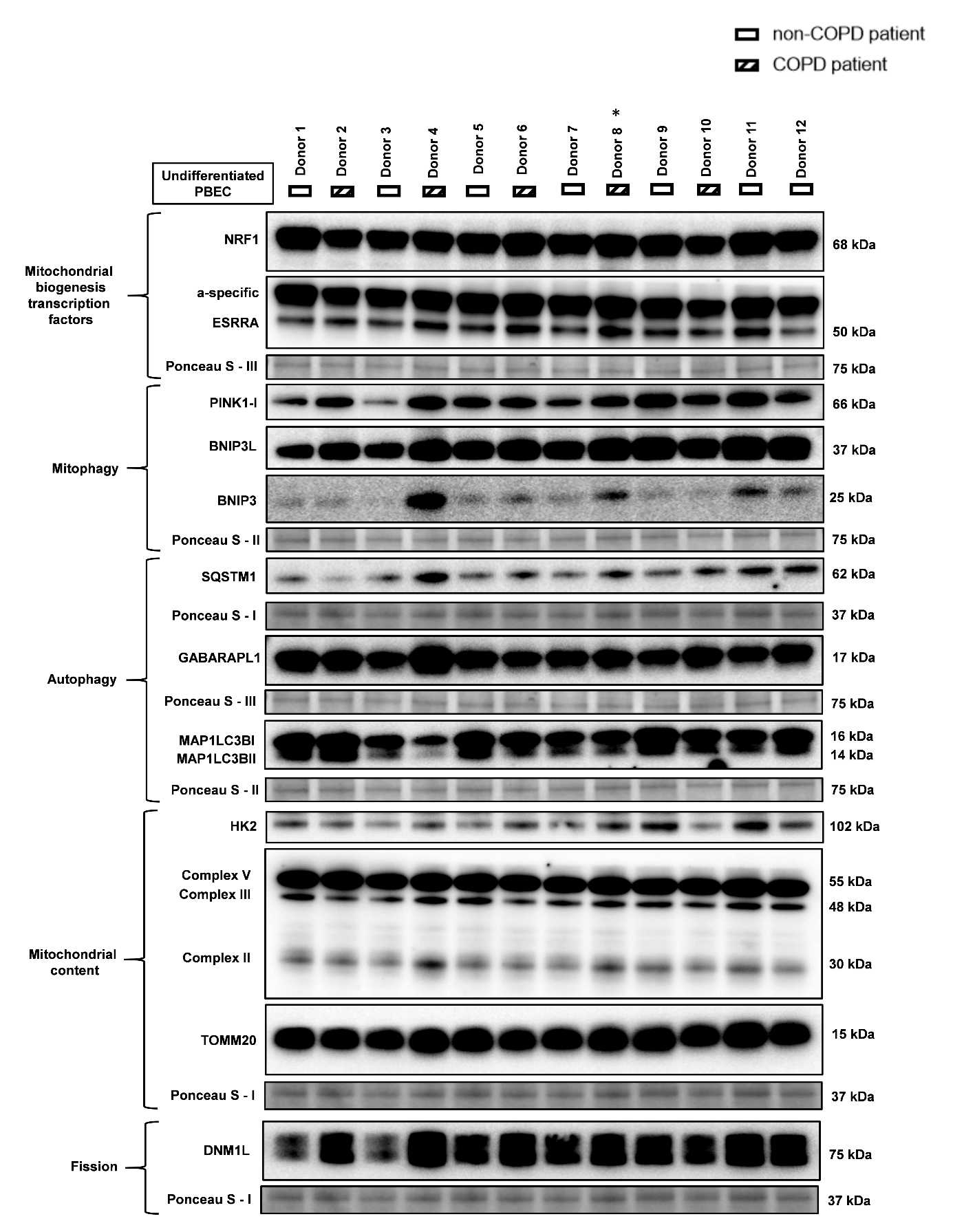
**

**Figure S2: Abundance of proteins involved in mitochondrial turnover and metabolism in undifferentiated PBEC from COPD patients.** Protein abundance of transcription factors involved in mitochondrial biogenesis, as well as regulators associated with mitophagy, autophagy, mitochondrial content and fission in undifferentiated primary bronchial epithelial cells (PBEC) from COPD patients (n=4) relative to non-COPD patients (n=7). A pool of quintuplicates per donor was analysed. All western blot images of the target proteins along with the corresponding normalization bands of the Ponceau S staining are shown of all patients/group. The Ponceau S band (37; 75 kDa) displayed is representative for the total Ponceau S staining per sample. Because several proteins of interest were detected on a similar gel, which is possible due to their difference in molecular mass, correction for total protein content/loading of these target proteins was based on the same Ponceau S staining. To specify, Gel I: HK2, DNM1L, OXPHOS, SQSTM1, TOMM20; Gel II: BNIP3L, BNIP3, PINK1, MAP1LC3B; Gel III: NRF1, GABARAPL1, ESRRA. ^*^This donor was excluded post-analysis due to disease status (i.e., GOLDI *versus* only GOLDII patients included in the COPD patient group).

**
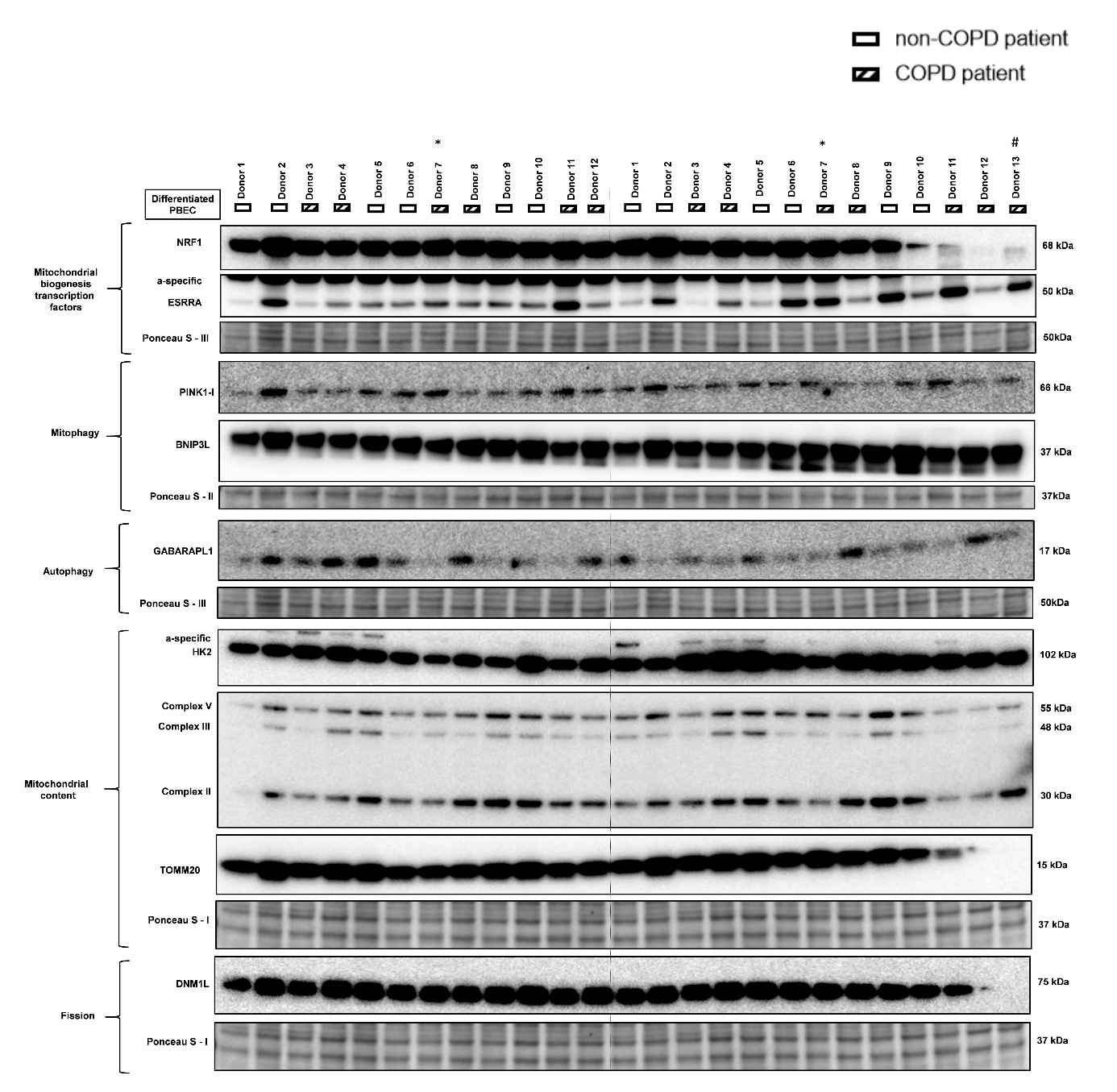
**

**Figure S3: Abundance of proteins involved in mitochondrial turnover and metabolism in differentiated PBEC from COPD patients.** Protein abundance of transcription factors involved in mitochondrial biogenesis, as well as regulators associated with mitophagy, autophagy, mitochondrial content and fission in differentiated primary bronchial epithelial cells (PBEC) from COPD patients (n=6) relative to non-COPD patients (n=6). Duplicates per donor were analysed (separated by the dotted line), with the exception for donor 13 as indicated by ^#^. The average of the duplicates/donor was used for calculation of the mean per group. All western blot images of the target proteins along with the corresponding normalization bands of the Ponceau S staining are shown of all patients/group. The Ponceau S band (37; 50 kDa) displayed is representative for the total Ponceau S staining per sample. Because several proteins of interest were detected on a similar gel, which is possible due to their difference in molecular mass, correction for total protein content/loading of these target proteins was based on the same Ponceau S staining. To specify, Gel I: HK2, DNM1L, OXPHOS, TOMM20; Gel II: BNIP3L, PINK1; Gel III: NRF1, GABARAPL1, ESRRA. Obviously, in case of technical difficulties, samples were excluded for analysis, for example detection of NRF1, TOMM20 and DNM1L protein levels was not possible for donors loaded in the last lanes of the gel/membrane. ^*^This donor (duplicates) was excluded post-analysis due to smoking behaviour (i.e., only non-smoker COPD patient, while other COPD patients included are ex-smokers).


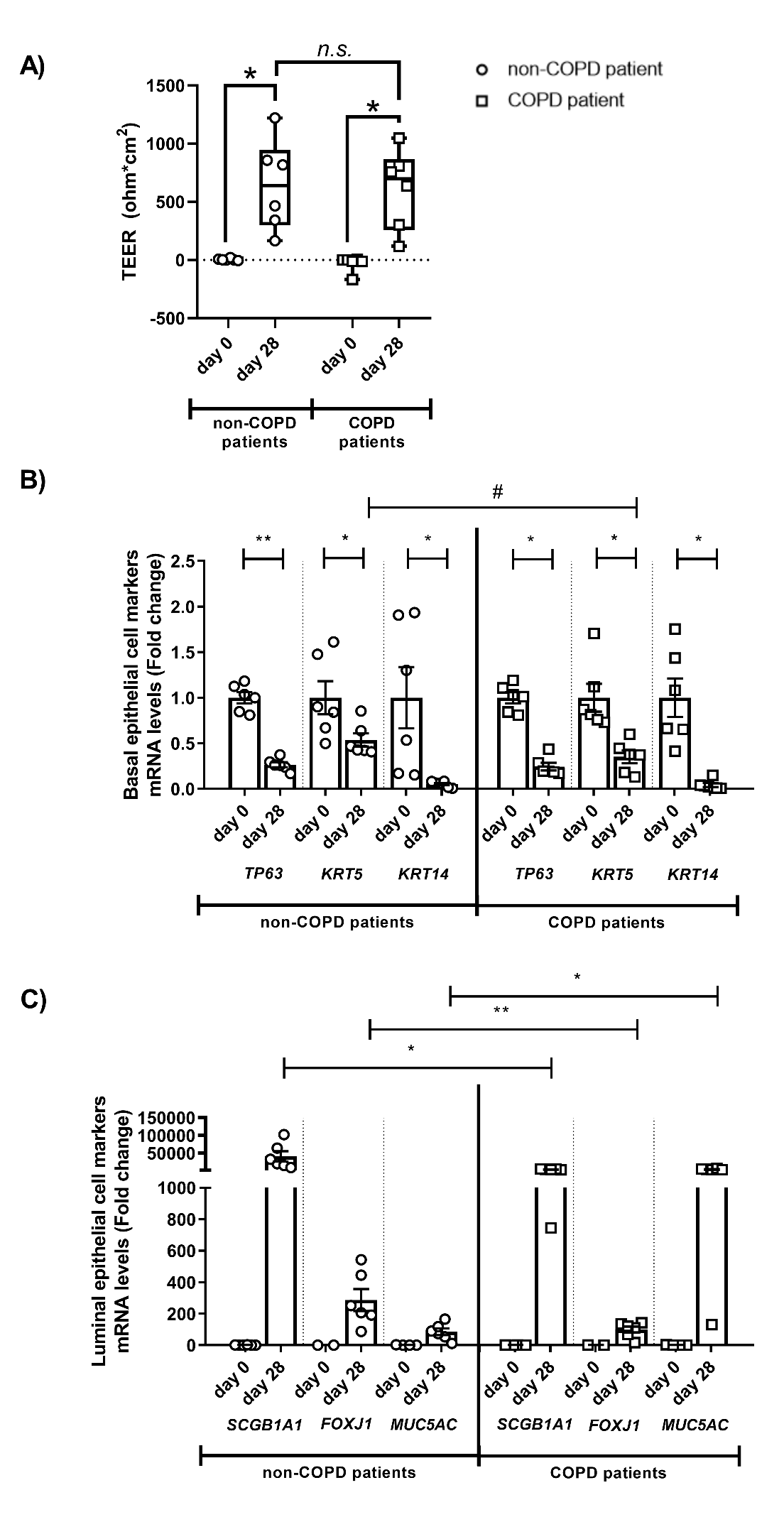


**Figure S4: Barrier formation and expression of cell type specific markers of differentiated PBEC from COPD patients.** Primary bronchial epithelial cells (PBEC) from non-COPD patients (n=6) or COPD patients (n=6) were proliferated (day 0) and differentiated at air-liquid interface (day 28). Validation and characterization of the differentiation of PBEC was conducted by transepithelial electrical resistance (TEER) as indication of barrier formation during differentiation on day 0 (undifferentiated) *versus* day 28 (fully differentiated). TEER data are presented as mean fold change of day 28 *versus* day 0 per patient group in box plots (min to max) **(A)**. Moreover, gene expression analysis was conducted of specific cell type markers of basal and luminal epithelial cells on day 0 (undifferentiated) *versus* day 28 (fully differentiated) of differentiation of the pseudostratified epithelium. To specify, expression of basal epithelial cell markers *TP63*, *KRT5* and *KRT14* **(B)** as well as expression of different cell populations present in the pseudostratified lung epithelium, respectively club cell secretory marker *SCGB1A1*, ciliogenesis marker *FOXJ1* and goblet cell differentiation marker *MUC5AC* **(C)** were presented. Individual subjects are represented by open circles (non-COPD patients) and open squares (all COPD patients). Gene expression data are presented as mean fold change of day 28 *versus* day 0 per patient group ± SEM. Statistical differences of TEER and gene expression data (basal epithelial cell markers) between day 28 (differentiated) relative to day 0 (undifferentiated) in separate patient groups were tested using a two-tailed paired parametric t-test, or in case of non-normal distribution a paired nonparametric Wilcoxon test. Moreover, differences of TEER and gene expression data between day 28 (differentiated) of non-COPD patients relative to COPD patients were tested using a two-tailed unpaired parametric t-test (normal distribution) or non-parametric Mann-Whitney test (non-normal distribution). Statistical significance is indicated as ^**^p<0.01, ^*^p<0.05 and a trend was indicated if ^#^p<0.1 compared to non-COPD patients.

**Table S1: Characteristics of patients: PBEC from non-COPD patients and COPD patients.** Characteristics of non-COPD patients or COPD patients from who primary bronchial epithelial cells (PBEC) were isolated and cultured submerged (undifferentiated) or differentiated at air-liquid interface. Data are presented as mean fold change of COPD patients relative to non-COPD patients ± SEM. FEV_1_: forced expiratory volume in first second; FVC: forced vital capacity. ^§^1 missing value; ^‡^2 missing values; †5 missing values. Statistical differences between COPD patients *versus* non-COPD patients were tested using a two-tailed unpaired parametric t-test (normal distribution), or an unpaired nonparametric Mann-Whitney test (non-normal distribution). Statistical significance is indicated as ^**^p<0.01 and a trend was indicated if ^#^p<0.1 compared to non-COPD patients.

| Characteristics | Undifferentiated PBEC | | Differentiated PBEC | |
| --- | --- | --- | --- | --- |
|  | **Non-COPD**  **patients** | **COPD**  **patients** | **Non-COPD**  **patients** | **COPD**  **patients** |
| Number of subjects | 7 | 4 | 6 | 6 |
| Male/Female | 3/4 | 4/0^#^ | 6/0 | 6/0 |
| Age (years) | 66.9 ± 2.8 | 61.8 ± 5.0 | 52.5 ± 4.1 | 65.0 ± 4.2^#^ |
| Body mass index | 27.5 ± 1.2 | 27.7 ± 2.1 | 28.0 ± 1.6 | 30.3 ± 1.6 |
| Smoking behaviour (Never/Ex-/Current/smoker) | 0/6/1 | 0/4/0 | 3/3/0 | 0/6/0^#^ |
| Pack-years (years) | 22.0 ± 2.0† | 45.0 ± 10.0^‡^ | 7.5 ± 4.0 | 43.8 ± 5.7^**^ |
| FEV_1_ (absolute) (L) | 2.8 ± 0.2 | 2.4 ± 0.2 | 3.9 ± 0.3 | 2.3 ± 0.4^**^ |
| FVC (absolute) (L) | - | - | 5.1 ± 0.3 | 4.6 ± 0.6 |
| FEV_1_/FVC (%) | 79.3 ± 3.4 | 60.2 ± 2.3^**^ | 77.3 ± 2.0 | 50.8 ± 4.0^**^ |
| Diffusing capacity of carbon monoxide (DLCO) SB (%) | 77.8 ± 4.0 | 79.0 ± 0.7^§^ | 92.2 ± 4.3 | 56.4 ± 8.4^**^ |
| Diffusing capacity of carbon monoxide (DLCO) VA (%) | 86.4 ± 4.9 | 91.0 ± 5.6^§^ | 96.6 ± 1.5 | 66.0 ± 7.6^**^ |

Table S2: Primer sequences of reference and target genes used for real time quantitative PCR analysis.

| **Gene** | **Sense primer (5’-3’)** | **Antisense primer (3’-5’)** |
| --- | --- | --- |
| **Reference genes** | | |
| *ACTB* | AAGCCACCCCACTTCTCTCTAA | AATGCTATCACCTCCCCTGTGT |
| *B2M* | CTGTGCTCGCGCTACTCTCTCTT | TGAGTAAACCTGAATCTTTGGAGTACGC |
| *PPIA* | CATCTGCACTGCCAAGACTGA | TTCATGCCTTCTTTCACTTTGC |
| *RPL13A* | CCTGGAGGAGAAGAGGAAAGAGA | TTGAGGACCTCTGTGTATTTGTCAA |
| **Target genes** | | |
| *BNIP3* | AGCGCCCGGGATGCA | CCCGTTCCCATTATTGCTGAA |
| *BNIP3L* | CTGCGAGGAAAATGAGCAGTCTCT | GCCCCCCATTTTTCCCATTG |
| *Citrate synthase* | GATGTGTCAGATGAGAAGTTACGAGACT | TGGCCATAGCCTGGAACAA |
| *COXII* | ACCTGCGACTCCTTGACGTT | GGGGGCTTCAATCGGGAGTA |
| *COXIII* | CGGCATCTACGGCTCAACAT | TGAGGAAAGTTGAGCCAATAATGAC |
| *COXIV* | CCATGGATGAGAAAGTCGAGT | CGTTCGAGCCCCTGTTCA |
| *CYC1* | GAGCACGACCATCGAAAACG | CGATATGCCAGCTTCCGACT |
| *CYTB* | ACCCCCTAGGAATCACCTCC | GCCTAGGAGGTCTGGTGAGA |
| *DNM1L* | CGACTCATTAAATCATATTTTCTCATTGTCAG | TGCATTACTGCCTTTGGCACACT |
| *ESRRA* | TGCTGCTCACGCTACCGCTC | TCGAGCATCTCCAAGAACAGC |
| *FIS1* | CCTGGTGCGGAGCAAGTACAA | TCCTTGCTCCCTTTGGGCAG |
| *FOXJ1* | TCGTATGCCACGCTCATCTG | CTTGTAGATGGCCGACAGGG |
| *FUNDC1* | GAAACGAGCGAACAAAGCAG | GCAAAAAGCCTCCCACAAAT |
| *GABARAPL1* | ATCGGAAAAAGGAAGGAGAAAAGATC | CAGGCACCCTGGCTTTTGG |
| *HADHA* | TGGCTTCCCGCCTTGTC | TGGAGCCGGTCCACTATCTTC |
| *HK2* | GTAAATACAGTGGATCTCAATCTTCGGG | CAAGGATTTGAGATGATTCGCTATTCA |
| *KRT5* | GGAGTTGGACCAGTCAACATC | TGGAGTAGTAGCTTCCACTGC |
| *KRT14* | TGGATCGCAGTCATCCAGAG | ATCGTGCACATCCATGACCT |
| *MAP1LC3A* | CCTGGACAAGACCAAGTTTTTG | GTCTTTCTCCTGCTCGTAGATG |
| *MAP1LC3B* | ACCATGCCGTCGGAGAAGAC | TCTCGAATAAGTCGGACATCTTCTACTCT |
| *MFN1* | CTGAGGATGATTGTTAGCTCCACG | CAGGCGAGCAAAAGTGGTAGC |
| *MFN2* | TGGACCACCAAGGCCAAGGA | TCTCGCTGGCATGCTCCAC |
| *MUC5AC* | AGCAGGGTCCTCATGAAGGTGGAT | AATGAGGACCCCAGACTGGCTGAA |
| *NDUFB3* | TCAGATTGCTGTCAGACATGG | TGGTGTCCCTTCTATCTTCCA |
| *NRF1* | AGGAACACGGAGTGACCCAA | TATGCTCGGTGTAAGTAGCCA |
| *NRF2* | CTCACCTGGGAACAGAACAGGAA | ACCCAAGAAATGCAGTCTCGAGC |
| *OPA1* | TACCAAAGGCATTTTGTAGATTCTGAGTT | GCATGCGCTGTATACGCCAA |
| *PDK4* | CCTGTGAGACTCGCCAACA | TCCACCAAATCCATCAGGCTC |
| *PINK1* | GAAAGCCGCAGCTACCAAGA | AGCACATTTGCGGCTACTCG |
| *PPARA* | CAGAACAAGGAGGCGGAGGTC | AGGTCCAAGTTTGCGAAGC |
| *PPARD* | TGACCAAAAAGAAGGCCCGC | GTCGTGGATCACAAAGGGCG |
| *PPARGC1A_1.0* | ATGCCTTTAGATGTGAGCTAACAGTAGGTAATGA | CGTACAGCCATCAAAAAGGGACAC |
| **Gene** | **Sense primer (5’-3’)** | **Antisense primer (3’-5’)** |
| *PPARGC1A_2.0* | AAGCCACTACAGACACCGC | TCGTAGCTGTCATACCTGGG |
| *PPARGC1B* | GGCGCTTTGAAGTGTTTGGTGA | TGATGAAGCCGTACTTCTCGCCT |
| *PPRC1* | GCCCTTTGATCTCTGCTTTGGG | AAGTCTTCCCGGTTGGAGTCAAG |
| *PRKN* | GGTTTGCCTTCTGCCGGGAATG | CTTTCATCGACTCTGTAGGCCTG |
| *SCGB1A1* | TTCAGCGTGTCATCGAAACCC | ACAGTGAGCTTTGGGCTATTTTT |
| *SQSTM1* | GGTGCACCCCAATGTGATCT | CGCAGACGCTACACAAGTCG |
| *TFAM* | GAAAGATTCCAAGAAGCTAAGGGTGATT | TCCAGTTTTCCTTTACAGTCTTCAGCTTTT |
| *TP63* | CCCGTTTCGTCAGAACACAC | CATAAGTCTCACGGCCCCTC |

**Abbreviations:** *ACTB*: Actin B, *B2M*: Beta-2 Microglobulin, *PPIA*: Peptidylprolyl Isomerase A, *RPL13A*: Ribosomal Protein L13A, *BNIP3*: BCL2 Interacting Protein 3, *BNIP3L*: BCL2 Interacting Protein 3-Like, *COXII*: Cytochrome C Oxidase Subunit II, *COXIII*: Cytochrome C Oxidase Subunit III, *COXIV*: Cytochrome C Oxidase Subunit 4I1 , *CYC1*: Cytochrome C-1, *CYTB*: Mitochondrially Encoded Cytochrome B, *DNM1L*: Dynamin 1-Like, *ESRRA*: Estrogen Related Receptor, Alpha, *FIS1*: Fission, Mitochondrial 1, *FOXJ1*: Forkhead Box J1, *FUNDC1*: FUN14 Domain Containing 1, *GABARAPL1*: GABA type A Receptor Associated Protein Like 1, *HADHA*: Hydroxyacyl-CoA Dehydrogenase Trifunctional Multienzyme Complex Subunit Alpha, *HK2*: Hexokinase 2, *KRT5*: Keratin 5, *KRT14*: Keratin 14, *MAP1LC3A*: Microtubule-Associated Protein 1 Light Chain 3 Alpha, *MAP1LC3B*: Microtubule-Associated Protein 1 Light Chain 3 Beta, *MFN1*: Mitofusin 1, *MFN2*: Mitofusin 2, *MUC5AC*: Mucin 5AC, Oligomeric Mucus/Gel-Forming, *NDUFB3*: NADH:Ubiquinone Oxidoreductase Subunit B3, *NRF1*: Nuclear Respiratory Factor 1, *NRF2*: Nuclear Respiratory Factor 2, *OPA1*: OPA1, Mitochondrial Dynamin Like GTPase, *PDK4*: Pyruvate Dehydrogenase Kinase 4, *PINK1*: PTEN Induced Kinase 1, *PPARA*: Peroxisome Proliferator Activated Receptor Alpha, *PPARD*: Peroxisome Proliferator Activated Receptor Delta, *PPARGC1A*: Peroxisome Proliferator-Activated Receptor Gamma, Coactivator 1 Alpha, *PPARGC1B*: Peroxisome Proliferator-Activated Receptor Gamma, Coactivator 1 Beta, *PPRC1*: Peroxisome Proliferator-Activated Receptor Gamma, Coactivator-Related 1, *PRKN:* Parkin RBR E3 Ubiquitin Protein Ligase, *SCGB1A1*: Secretoglobin Family 1a Member 1, *SQSTM1*: Sequestosome 1, transcript variant 1/4, *TFAM*: Transcription Factor A, Mitochondrial, *TP63*: Tumor Protein P63

Table S3: Primary and secondary antibodies used for western blotting

| **Target** | **RRID** | **Company** | **Product number** | **Dilution** |
| --- | --- | --- | --- | --- |
| **Primary Antibodies** | | | |  |
| BNIP3 | AB_2259284 | Cell Signaling Technology | Cat# 3769S | 1:1000 |
| BNIP3L | AB_2688036 | Cell Signaling Technology | Cat# 12396 | 1:1000 |
| DNM1L | AB_10950498 | Cell Signaling Technology | Cat# 8570 | 1:1000 |
| ESRRA | AB_1523580 | Abcam | Cat# ab76228 | 1:1000 |
| FUNDC1 | AB_10609242 | Santa Cruz Biotechnology | Cat# sc-133597 | 1:500 |
| GABARAPL1 | AB_2294415 | Proteintech Group | Cat# 11010-1-AP | 1:1000 |
| HK2 | AB_2232946 | Cell Signaling Technology | Cat# 2867 | 1:1000 |
| MAP1LC3B | AB_915950 | Cell Signaling Technology | Cat# 2775 | 1:1000 |
| NRF1 | AB_2154534 | Abcam | Cat# ab55744 | 1:1000 |
| OXPHOS  antibody cocktail | AB_2629281 | MitoScience LLC | Cat# MS604 | 1:1000 |
| PINK1 | AB_10127658 | Novus Biologicals | Cat# BC100-494 | 1:2000 |
| PRKN | AB_2159920 | Cell Signaling Technology | Cat# 4211 | 1:1000 |
| SQSTM1 | AB_10624872 | Cell Signaling Technology | Cat# 5114 | 1:1000 |
| TFAM | AB_10682431 | Millipore | Cat# DR1071 | 1:1000 |
| TOMM20 | AB_2716623 | Abcam | Cat# Ab186734 | 1:1000 |
| **Secondary Antibodies** | | | |  |
| Goat Anti-Mouse IgG Antibody | AB_2827937 | Vector Laboratories | Cat#BA-9200 | 1:10000 |
| Goat Anti-Rabbit IgG Antibody | AB_2313606 | Vector Laboratories | Cat#BA-1000 | 1:10000 |
| Goat Anti-Rabbit IgG Antibody | AB_2099233 | Cell Signaling Technology | Cat#7074S | 1:10000 |

**Abbreviations:** BNIP3: BCL2 Interacting Protein 3, BNIP3L: BCL2 Interacting Protein 3-Like, DNM1L: Dynamin-1-Like, ESRRA: Estrogen Related Receptor, Alpha, FUNDC1: FUN14 Domain Containing 1, GABARAPL1: GABA Type A Receptor Associated Protein Like 1, HK2: Hexokinase 2, MAP1LC3 (or LC3B): Microtubule-Associated Protein 1 Light Chain 3 Beta, NRF1: Nuclear Respiratory Factor 1, OXPHOS antibody cocktail: oxidative phosphorylation antibody cocktail (containing NDUFB8: NADH:Ubiquinone Oxidoreductase Subunit B8, SDHB: Succinate Dehydrogenase Complex Iron Sulfur Subunit B, UQCRC2: Ubiquinol-Cytochrome C Reductase Core Protein 2, MT-COI: Mitochondrially Encoded Cytochrome C Oxidase I, ATP5F1A: ATP Synthase F1 Subunit Alpha), PINK1: PTEN Induced Kinase 1, PRKN: Parkin RBR E3 Ubiquitin Protein Ligase, SQSTM1: Sequestosome 1, TFAM: Transcription factor A, Mitochondrial, TOMM20: Translocase Of Outer Mitochondrial Membrane 20

**Table S4. Gene expression of markers involved in mitochondrial turnover and metabolism in PBEC from COPD patients.** mRNA levels of constituents involved in the regulation of mitochondrial turnover and mitochondrial metabolic pathways were analysed in undifferentiated primary bronchial epithelial cells (PBEC) (triplicates/donor) or differentiated PBEC at air-liquid interface (duplicates/donor) from non-COPD patients or COPD patients. Donors included in the undifferentiated PBEC study are current or ex-smoker non-COPD patients (n=7), while COPD patients included are ex-smokers (n=4). Donors included in the differentiated PBEC study are never- or ex-smoker non-COPD patients (n=6), while COPD patients included are all ex-smokers (n=6). The average of triplicates/donor (undifferentiated PBEC) or duplicates/donor (differentiated PBEC) was used for calculation of the mean per group. Data are presented as mean fold change of COPD patients relative to non-COPD patients ± SEM. Statistical differences between COPD patients *versus* non-COPD patients were tested using a two-tailed unpaired parametric t-test, or in case of non-normal distribution an unpaired nonparametric Mann-Whitney test. Statistical significance is indicated as ^*^p<0.05 or ^#^p<0.1 compared to non-COPD patients.

| mRNA expression | Undifferentiated PBEC | | | | Differentiated PBEC | | |
| --- | --- | --- | --- | --- | --- | --- | --- |
|  | **Non-COPD**  **patients**  **N=7** | | **COPD**  **patients**  **N=4** | **Non-COPD**  **patients**  **N=6** | | **COPD**  **patients**  **N=6** | |
| Mitochondrial biogenesis |  |  | | |  | |  |
| *PPARGC1A* | - | - | | | 1.0 ± 0.28 | | 0.96 ± 0.14 |
| *PPARGC1B* | 1.0 ± 0.07 | 0.78 ± 0.03^*^ | | | 1.0 ± 0.08 | | 0.73 ± 0.08^*^ |
| *PPRC1* | 1.0 ± 0.09 | 0.91 ± 0.05 | | | 1.0 ± 0.10 | | 0.91 ± 0.07 |
| Mitochondrial biogenesis transcription factors |  |  | | |  | |  |
| *PPARA* | 1.0 ± 0.08 | 0.86 ± 0.03 | | | 1.0 ± 0.15 | | 0.97 ± 0.10 |
| *ESRRA* | 1.0 ± 0.07 | 0.98 ± 0.05 | | | - | | - |
| *NRF2* | 1.0 ± 0.05 | 0.94 ± 0.04 | | | 1.0 ± 0.07 | | 0.83 ± 0.05^#^ |
| *TFAM* | 1.0 ± 0.06 | 0.95 ± 0.06 | | | 1.0 ± 0.10 | | 0.95 ± 0.07 |
| Ubiquitin-mediated mitophagy |  |  | | |  | |  |
| *PINK1* | 1.0 ± 0.09 | 1.0 ± 0.04 | | | 1.0 ± 0.17 | | 0.95 ± 0.08 |
| *PRKN* | 1.0 ± 0.25 | 1.1 ± 0.18 | | | 1.0 ± 0.31 | | 0.82 ± 0.10 |
| Receptor-mediated mitophagy |  |  | | |  | |  |
| *BNIP3* | 1.0 ± 0.09 | 1.0 ± 0.08 | | | 1.0 ± 0.05 | | 0.90 ± 0.08 |
| *BNIP3L* | 1.0 ± 0.07 | 1.2 ± 0.10 | | | 1.0 ± 0.07 | | 0.93 ± 0.09 |
| *FUNDC1* | 1.0 ± 0.03 | 0.85 ± 0.12 | | | 1.0 ± 0.05 | | 1.1 ± 0.09 |
| Autophagy |  |  | | |  | |  |
| *SQSTM1* | 1.0 ± 0.11 | 0.96 ± 0.06 | | | 1.0 ± 0.09 | | 0.98 ± 0.08 |
| *GABARAPL1* | 1.0 ± 0.11 | 1.0 ± 0.06 | | | 1.0 ± 0.16 | | 1.2 ± 0.14 |
| *MAP1LC3A* | 1.0 ± 0.15 | 0.93 ± 0.10 | | | 1.0 ± 0.31 | | 0.67 ± 0.08 |
| *MAP1LC3B* | 1.0 ± 0.15 | 0.95 ± 0.07 | | | 1.0 ± 0.06 | | 0.95 ± 0.09 |
| Electron transport chain |  |  | | |  | |  |
| *NDUFB3 (COXI)* | - | - | | | 1.0 ± 0.11 | | 0.85 ± 0.06 |
| *CYC1 (COXIII)* | - | - | | | 1.0 ± 0.09 | | 0.84 ± 0.03 |
| *COXIII (COXIV)* | 1.0 ± 0.06 | 1.0 ± 0.09 | | | - | | - |
| Tricarboxylic acid cycle |  |  | | |  | |  |
| *Citrate synthase* | 1.00 ± 0.05 | 0.96 ± 0.02 | | | 1.0 ± 0.16 | | 0.99 ± 0.12 |
| Glycolysis |  |  | | |  | |  |
| *HK2* | 1.0 ± 0.09 | 0.94 ± 0.04 | | | 1.0 ± 0.11 | | 1.1 ± 0.09 |
| Fatty acid β-oxidation |  |  | | |  | |  |
| *HADH* | 1.00 ± 0.05 | 0.92 ± 0.05 | | | 1.0 ± 0.11 | | 0.88 ± 0.05 |
| Fission |  |  | | |  | |  |
| *DNM1L* | 1.0 ± 0.05 | 0.96 ± 0.06 | | | 1.0 ± 0.07 | | 0.91 ±0.06 |
| *FIS1* | 1.0 ± 0.04 | 0.98 ± 0.04 | | | 1.0 ± 0.08 | | 0.87 ± 0.09 |
| Fusion |  |  | | |  | |  |
| *MFN1* | 1.0 ± 0.07 | 0.91 ± 0.05 | | | 1.0 ± 0.10 | | 0.91 ± 0.07 |
| *MFN2* | - | - | | | 1.0 ± 0.08 | | 0.94 ± 0.13 |
| *OPA1* | 1.0 ± 0.06 | 0.90 ± 0.04 | | | 1.0 ± 0.04 | | 0.83 ± 0.04^*^ |

**Table S5. Protein expression of markers involved in mitochondrial turnover and metabolism in PBEC from COPD patients.** Protein levels of constituents involved in the regulation of mitochondrial turnover and mitochondrial metabolic pathways were analysed in undifferentiated primary bronchial epithelial cells (PBEC) (duplicates/donor) or differentiated PBEC at air-liquid interface (pool of quintuplicates/donor) from non-COPD patients or COPD patients. Donors included in the undifferentiated PBEC study are current or ex-smoker non-COPD patients (n=5-7), while COPD patients included are ex-smokers (n=3-4). Donors included in the differentiated PBEC study are never- or ex-smoker non-COPD patients (n=6), while COPD patients included are all ex-smokers (n=5-6). The average of duplicates/donor was used for calculation of the mean per group for undifferentiated PBEC. Data are presented as mean fold change of COPD patients relative to non-COPD patients ± SEM. Statistical differences between COPD patients *versus* non-COPD patients were tested using a two-tailed unpaired parametric t-test, or in case of non-normal distribution an unpaired nonparametric Mann-Whitney test. A trend is indicated as ^#^p<0.1 compared to non-COPD patients.

| Protein expression | Undifferentiated PBEC | | | | Differentiated PBEC | | |
| --- | --- | --- | --- | --- | --- | --- | --- |
|  | **Non-COPD**  **patients**  **N=5-7** | | **COPD**  **patients**  **N=3-4** | **Non-COPD**  **patients**  **N=6** | | **COPD**  **patients**  **N=5-6** | |
| Mitochondrial biogenesis transcription factors |  |  | | |  | |  |
| ESRRA | 1.0 ± 0.10 | 0.96 ± 0.08 | | | 1.0 ± 0.23 | | 1.4 ± 0.55 |
| NRF1 | 1.0 ± 0.09 | 0.71 ± 0.11^#^ | | | 1.0 ± 0.07 | | 0.93 ± 0.06 |
| Ubiquitin-mediated mitophagy |  |  | | |  | |  |
| PINK1-I | 1.0 ± 0.25 | 1.1 ± 0.06 | | | 1.0 ± 0.16 | | 0.82 ± 0.11 |
| Receptor-mediated mitophagy |  |  | | |  | |  |
| BNIP3 | 1.0 ± 0.19 | 2.2 ± 1.3 | | | - | | - |
| BNIP3L | 1.0 ± 0.19 | 0.96 ± 0.14 | | | 1.0 ± 0.08 | | 0.93 ± 0.08 |
| Autophagy |  |  | | |  | |  |
| SQSTM1 | 1.0 ± 0.07 | 1.3 ± 0.52 | | | - | | - |
| GABARAPL1 | 1.0 ± 0.07 | 1.3 ± 0.37 | | | 1.0 ± 0.09 | | 1.0 ± 0.09 |
| Ratio MAP1LC3BII/I | 1.0 ± 0.04 | 1.0 ± 0.13 | | | - | | - |
| Electron transport chain |  |  | | |  | |  |
| SDHB (COXII) | 1.0 ± 0.10 | 1.1 ± 0.25 | | | 1.0 ± 0.22 | | 0.96 ± 0.18 |
| UQCRC2 (COXIII) | 1.0 ± 0.10 | 0.88 ± 0.24 | | | 1.0 ± 0.23 | | 0.65 ± 0.26 |
| ATP5A (COXV) | 1.0 ± 0.10 | 1.1 ± 0.16 | | | 1.0 ± 0.15 | | 0.67 ± 0.06^#^ |
| Translocase of outermembrane |  |  | | |  | |  |
| TOMM20 | 1.0 ± 0.09 | 0.97 ± 0.17 | | | 1.0 ± 0.06 | | 0.93 ± 0.08 |
| Glycolysis |  |  | | |  | |  |
| HK2 | 1.0 ± 0.09 | 0.93 ± 0.14 | | | 1.0 ± 0.11 | | 1.1 ± 0.10 |
| Fission |  |  | | |  | |  |
| DNM1L | 1.0 ± 0.13 | 2.0 ± 0.68 | | | 1.0 ± 0.09 | | 0.90 ± 0.08 |
